# Induction of Progenitor Exhausted Tissue-Resident Memory CD8^+^ T Cells Upon *Salmonella* Typhi Porins Adjuvant Immunization Correlates With Melanoma Control and Anti-PD-1 Immunotherapy Cooperation

**DOI:** 10.3389/fimmu.2020.583382

**Published:** 2020-11-06

**Authors:** Ricardo A. León-Letelier, Daniel I. Castro-Medina, Oscar Badillo-Godinez, Araceli Tepale-Segura, Enrique Huanosta-Murillo, Cristina Aguilar-Flores, Saraí G. De León-Rodríguez, Alejandra Mantilla, Ezequiel M. Fuentes-Pananá, Constantino López-Macías, Laura C. Bonifaz

**Affiliations:** ^1^ Unidad de Investigación Médica en Inmunoquímica, UMAE Hospital de Especialidades, Centro Médico Nacional Siglo XXI, Instituto Mexicano del Seguro Social, Mexico City, Mexico; ^2^ Posgrado en Ciencias Biomédicas, Universidad Nacional Autónoma de México, Mexico City, Mexico; ^3^ Centro de Investigación Sobre Enfermedades Infecciosas, Instituto Nacional de Salud Pública, Cuernavaca, Mexico; ^4^ Posgrado en Ciencias Biológicas, Universidad Nacional Autónoma de México, Mexico City, Mexico; ^5^ Servicio de Patología, Hospital de Oncología, Centro Médico Nacional Siglo XXI, Instituto Mexicano del Seguro Social, Mexico City, Mexico; ^6^ Unidad de Investigación en Virología y Cáncer, Hospital Infantil de México Federico Gómez, Mexico City, Mexico

**Keywords:** melanoma, immunotherapy, adjuvant, tissue-resident memory T cells, progenitor exhausted T cells, anti-PD-1 Ab

## Abstract

Immunotherapy has improved the clinical response in melanoma patients, although a relevant percentage of patients still cannot be salvaged. The search for the immune populations that provide the best tumor control and that can be coaxed by immunotherapy strategies is a hot topic in cancer research nowadays. Tumor-infiltrating TCF-1^+^ progenitor exhausted CD8^+^ T cells seem to grant the best melanoma prognosis and also efficiently respond to anti-PD-1 immunotherapy, giving rise to a TIM-3^+^ terminally exhausted population with heightened effector activity. We tested Porins from *Salmonella* Typhi as a pathogen associated molecular pattern adjuvant of natural or model antigen in prophylactic and therapeutic immunization approaches against murine melanoma. Porins induced protection against melanomas, even upon re-challenging of tumor-free mice. Porins efficiently expanded IFN-γ-producing CD8^+^ T cells and induced central and effector memory in lymph nodes and tissue-resident (Trm) T cells in the skin and tumors. Porins induced TCF-1^+^ PD-1^+^ CD8^+^ Trm T cells in the tumor stroma and the presence of this population correlated with melanoma growth protection in mice. Porins immunization also cooperated with anti-PD-1 immunotherapy to hamper melanoma growth. Importantly, the potentially protective Trm populations induced by Porins in the murine model were also observed in melanoma patients in which their presence also correlated with disease control. Our data support the use of cancer vaccination to sculpt the tumor stroma with efficient and lasting Trm T cells with effector activities, highlighting the use of Porins as an adjuvant. Furthermore, our data place CD8^+^ Trm T cells with a progenitor exhausted phenotype as an important population for melanoma control, either independently or in cooperation with anti-PD-1 immunotherapy.

## Introduction

Melanoma is the most aggressive form of skin cancer, and even though it only accounts for 1%, it is the cause of the majority of the skin cancer-related deaths. The incidence rate of melanoma has risen rapidly in the last three decades, but its mortality has decreased (1), perhaps because of the immunotherapy strategies that have revolutionized melanoma treatment. Since immunotherapy aims to strengthen anti-tumoral activities, successful responses depend on the state of the immune system and in the density of tumor-infiltrating lymphocytes (TILs), particularly of T cells (2). Thus, melanomas often do not respond to immunotherapy due to lack of T cell infiltration, or because T cells have acquired dysfunctional phenotypes (3). Dissecting the specific T cell subsets responsible for anti-tumoral effector functions and understanding the mechanisms that lead them to mediate healthy immunotherapy responses are central objectives of the revolutionary way in which we treat cancer nowadays.

Immunotherapy has helped to better understand the tumor stroma population dynamics pointing out to T cells with and without anti-tumor activity. Long-lasting immune anti-tumoral responses depend on formation of memory T cells, central (Tcm), effector (Tem), and tissue-resident memory (Trm). Trm T cells are long-lived in tissues that have undergone antigenic challenge. Indeed, solid tumors are often infiltrated by CD8^+^ Trm T cells that are critical to eliminate tumor cells (4, 5). Trm T cells are characterized by the stable expression of CD69 and CD103, which signal for retention in tissues and for rapid effector function (4, 6). However, not all T cells infiltrating the tumor stroma seem to be able to display effector functions, with some populations exhibiting exhausted phenotypes, and Trm T cells can express high levels of PD-1 (7). Exhausted populations were first defined because of expression of this check-point marker, and were originally associated with T cell dysfunction due to chronic antigen stimulation (8). However, based on murine models of chronic viral infection, we are now beginning to understand the different PD-1^+^ subsets conforming the exhausted T cells, for instance, those recognized as “progenitor exhausted” with a TCF-1^+^ PD-1^+^ phenotype, and the “terminally exhausted” TIM-3^+^ PD-1^+^ (9, 10). The relevance to separate these subtypes lies in that only the progenitor exhausted T CD8^+^ population proliferates after anti-PD-1 therapy, resulting in immune control of the chronic viral infection (11–13). Recently, these two subtypes of exhausted CD8^+^ T cells have been also identified in the infiltrate of murine melanoma (9, 14), and in melanoma, kidney and lung cancer patients (14–16). Similar to the chronic viral model, only the progenitor exhausted CD8^+^ cells proliferated in response to anti-PD-1 therapy (9). Indeed, a human equivalent population of exhausted CD8^+^ TCF-7^+^ (the human ortholog of TCF-1) T cells have been found to predict positive responses to anti-PD-1 immunotherapy and enhanced survival of melanoma patients (17). Although this progenitor exhausted T cells do not display effector functions upon PD-1 blockade, they differentiate into terminally exhausted T cells with potent anti-tumor activities, such as IFN-γ secretion (18, 19). Thus, while both progenitor and terminally exhausted phenotypes are essential, the former seems to sense and trigger anti-PD-1-induced T cell effector functions (9, 10).

One mechanism to induce anti-tumor immune responses and provide long-lasting protection against relapse is by fostering T cell responses through immunization with cancer vaccines, a strategy that potentially could also synergize with immune check-point inhibitors. To achieve an efficient immunization, it is crucial not only to choose the proper antigen, but also a suitable adjuvant (20). Some adjuvants based on pathogen associated molecular patterns (PAMP) have shown protection against tumor growth and enhancement of the immune check-point inhibitors efficacy (21–24), but the synergic mechanisms of these adjuvants with the inhibitors is not completely understood. One adjuvant with a potent effect in a melanoma model is the nontoxic Cholera B subunit (CTB), a non-classical PAMP that activates the immune cells through NLRP3 and FcRγ-CARD9 (25, 26). We have previously demonstrated that an intradermal (i.d.) immunization with CTB and a model antigen promotes an efficient IFN-γ^+^ CD4^+^ T-cell response (27), that increase the survival of mice challenged with melanoma associated with a CD4^+^ Trm response (28–30). However, the participation of CD8^+^ T cells was not evaluated.

We have also studied the adjuvant capacity of highly purified outer membrane proteins (Porins) from Salmonella enterica serovar Typhi (*S.* Typhi). Porins are a classical PAMP that induce a potent antibody and T cell specific immune response in mice and humans (31, 32). We have previously reported that Porins were capable to increase the expression of the costimulatory molecules CD86 and CD40 on dendritic cells (DCs) through TLR2 and TLR4 (33). Moreover, Porins were also able to induce Porin-specific CD8^+^ and CD4^+^ T cells and antigen-specific CD4 response when used as an adjuvant (31, 34–36). Nevertheless, the capacity of Porins as an adjuvant to induce antigen-specific CD8s has not been tested, nor has the Porins adjuvant strength in a tumor model.

Different skin immunization strategies are able to induce long-lasting CD8^+^ Trm anti-tumoral responses (37, 38). The capacity of Porins to activate DCs and T cell responses points it out as a good candidate to test in a tumor model. In this study, we designed different immunization strategies aiming to induce Trm responses able to control melanoma initiation and progression. We observed that Porins gave rise to a CD8^+^ Trm PD-1^+^ T cell population that also express TCF-1, whose generation marked mice with better control of melanoma growth. Although it is known that Trm with effector functions can be induced in the tumor (38), our data argue that the choice of adjuvant in cancer vaccination can lead to formation of progenitor exhausted CD8^+^ Trm T cells, and that formation of this population correlates with the capacity to control melanoma cells independently and in cooperation with anti-PD-1 immunotherapy. Remarkably, the populations induced by Porins immunization were also identified in human melanoma patients associated with disease control.

## Materials and Methods

### Mice

Wild-type C57BL/6 mice were obtained from Unidad de Medicina Experimental, UNAM animal facility. The OT-IxCD45.1^+^ mice were kindly provided by Dr. J.C. Crispín, Instituto Nacional de Ciencias Médicas y Nutrición Salvador Zubirán (INCMNSZ), and OT-IIxCD45.1^+^ were kindly provided by Dr. G. Soldevila, Instituto de Investigaciones Biomédicas, UNAM animal facility. All mice were male and age (8–12 weeks)-matched. All animal experiments were performed following the Institutional Ethics Committee and the Mexican national regulations on animal care and experimentation (R-2015-785-008).

### Porins Purification

Porins were purified from *S.* Typhi 9,12, Vi:d. ATCC 9993 (Omp-C and Omp-F) using the method previously published (32). Briefly, *S.* Typhi was grown in glucose- supplemented minimal A medium and Porins were extracted from the bacteria using buffer with sodium dodecyl sulfate. Proteins were purified by molecular exclusion chromatography using a Sephacryl S-200 column. Chromatographically purified proteins were analyzed by sodium dodecyl sulfate-polyacrylamide gel electrophoresis. Lipopolysaccharide content was evaluated using a Limulus Amoebocyte Lysate Assay (Endosafe KTA, Charles River Endosafe Laboratories), and all batches used in the study were negative (detection limit, 0.2 ng LPS/mg protein). Western blot analysis using anti-LPS polyclonal sera confirmed that LPS was not detectable.

### Melanoma Culture and Tumor Challenge

B16-F10 or B16-F10-OVA (MO4) melanoma cells were cultured in DMEM containing 10% FBS, 0.1% penicillin/ streptomycin, 0.2% l-glutamine, 0.05% 2-mercaptoethanol, 0.01% sodium pyruvate, 0.1% HEPES, and 0.1% nonessential amino acids. Melanoma tumors were established by subcutaneous (s.c.) injection of 2.5 × 10^5^ cells in the left flank. The tumors width and length were measured using a caliper every 2 days starting at day 7. The tumor volume was calculated as 4/3π (1/2 width)^2^(1/2 length), in mm^3^. Tumor appearance was scored daily through manual palpation, and mice with no evidence of tumor by the end of the following period were scored as tumor-free.

### Prophylactic Immunization

For prophylactic experiments, mice were challenged with MO4 7 days after the immunization with 30 μg of ovalbumin (OVA) plus adjuvant: 10 μg of Porins or 10 μg of CTB (Sigma-Aldrich), injected s.c. in the left flank. As controls, mice were s.c. injected with OVA, Porins, CTB or PBS. For the re-challenge experiment, the tumor-free OVA + Porins immunized mice at day 28 were re-challenged with MO4 and followed up for 100 days from the first immunization. For the melanoma-associated antigen (MAAs) experiment, mice were injected with 70 nmol of the human/mouse recombinant peptides TRP-2 (AnaSpec) and gp100 (MyBioSource), with or without Porins, 10 and 3 days before challenge with the B16-F10 melanoma cells (39, 40).

### Therapeutic Immunization

For therapeutic experiments the MO4 challenge was 7 days prior the immunization (explained above) and followed up for 21 days. The therapeutic immunization was also executed with or without intraperitoneal (i.p.) injection of 100 μg anti-PD-1 monoclonal antibody (mAb) (BioLegend Cat# 114102, RRID:AB_313573), at days 19, 21, and 23, only for the immunized group, followed up for 28 days.

### Circulating Lymphocyte Depletion Experiment

OVA + Porins immunized mice received i.p. 100 μg of anti-CD8 mAb (TIB105, Clone 53-6.72, in house) or 250 μg of anti-CD4 mAb (GK1.5, in house) or 250 μg of control mAb (III-10, in house) as follows: 1 day before MO4 challenge, on the day of MO4 challenge and every 3 days after, with the last two injections 4 days apart, up to day 12 or 21, specified in each experiment. The OVA + Porins immunization was 7 or 28 days, or 28 days/boost before the MO4 challenge. At the end of the experiment, tumors and blood were harvested for Trm T cells and circulating lymphocyte identification, respectively.

### CD8^+^ and CD4^+^ OVA-Specific T Cell Enrichment and Adoptive Transfer

Skin-draining lymph nodes (SDLN), mesenteric lymph nodes and spleen were collected from OT-IxCD45.1^+^ and OT-IIxCD45.1^+^ mice and worked separately as described elsewhere (28). Briefly: recovered cells (see below) were incubated for 30 min on ice with homemade rat hybridoma supernatants, for OT-IxCD45.1^+^ T cells: against CD4, B cells, MHCII-expressing cells, macrophages and NK cells; for OTI-IIxCD45.1^+^ T cells: against CD8, B cells, MHCII-expressing cells, macrophages and NK cells. Next, cells were poured into Petri dishes previously coated with rat anti-IgG, and non-adherent cells were recovered for injection through the retro orbital vein. Congenic mice received 3 × 10^6^ OT-IxCD45.1^+^ cells and 3 × 10^6^ OT-IIxCD45.1^+^ cells intravenously (i.v.). After 24 h, anesthetized mice were i.d. immunized in both ears with 15 μg of OVA with the adjuvant: 5 μg Porins or 5 μg CTB.

### Skin and Skin-Draining Lymph Node Processing

Mice were sacrificed at 7 or 28 days post-immunization in the ear, to collect SDLN and skin. SDLN were macerated and filtered, and skin-infiltrating lymphocytes were obtained as previously described (28). Briefly: Skin cell suspensions were obtained by enzymatic digestion with Liberase TL and DNAse, then chopped and incubated under the same conditions. Next, enzymatic digestion was stopped, and cell suspensions were filtered followed by the addition of DNAse. Finally, cells were washed with PBS, counted and stained. SDLN cells were re-stimulated as previously reported (28). Briefly: Cells were incubated for 48 h with 1 mg/ml of SIINFEKL (InvivoGen) and OVA peptide 323–339 (InvivoGen), followed by cell stimulation cocktail plus protein transport inhibitor for 4 h at 37°C. Then the lymphocytes were collected, washed, and stained.

### Tumor Processing

At the end of the tumor growth experiments, tumors were harvested and divided in two fractions that were analyzed by immunofluorescence and flow cytometry. TILs were obtained with the method previously documented (21). Briefly: The tumor was chopped and incubated with Collagenase D and DNAse. Finishing the enzymatic reaction, the cell suspension was filtered, followed by the addition of DNAse. The lymphocyte interface from the centrifuged 40/90 Percoll solution was collected, washed, counted and stained.

### Flow Cytometry

Cells were stained with anti-CD45-PECy7 (BioLegend Cat# 157613, RRID:AB_2832559), and DAPI (ThermoFisher), mixed with CountBright absolute counting beads (ThermoFisher) and acquired for flow cytometry. Cell surface staining was performed first blocking with FACS and then adding the following antibodies: anti-CD45-PB (BioLegend Cat# 103126, RRID:AB_493535) or -PECy7, anti-CD45.1-Percp-Cy5.5 (BD Biosciences Cat# 560580, RRID:AB_1727489), anti-CD8-FITC (BioLegend Cat# 100705, RRID:AB_312744, Clone 53-6.70), -APC (BD Biosciences Cat# 553035, RRID:AB_398527) or -APC-Cy7 (BioLegend Cat# 100714, RRID:AB_312753), anti-CD4-APC-Cy7 (BioLegend Cat# 100526, RRID:AB_312727), anti-CD44-BV510 (BioLegend Cat# 103043, RRID:AB_2561391), anti-CD62L-PE-Cy7 (BioLegend Cat# 104418, RRID:AB_313103), anti-CD103-PECy7 (BioLegend Cat# 121426, RRID:AB_2563691), anti-CD69-PE (BioLegend Cat# 104507, RRID:AB_313110), anti-PD-1-APC (BioLegend Cat# 135209, RRID:AB_2251944), and anti-TIM-3-PE/Dazzle^TM^594 (BioLegend Cat# 134014, RRID:AB_2632738). LIVE/DEAD Fixable Aqua (Thermofisher) staining was included. To achieve intracellular staining, cell surface staining was first performed, followed by fixation and permeabilization using the intracellular fixation and permeabilization buffer set (Thermofisher), according to the manufacturer’s instructions. Intracellular staining included anti-IFN-γ-APC (BioLegend Cat# 505810, RRID:AB_315404) and anti-TCF-1/7-AF488 (Cell Signaling Technology Cat# 6444, RRID:AB_2797627). Cells were acquired in a BD FACSCanto II or BD FACSAria cytometer (Becton, Dickinson and company). Data obtained from the cytometer was analyzed with FlowJo software (Tree Star, Inc.).

### Mice Immunofluorescence Assay

Tumoral tissue was embedded in Tissue-Tek (Sakura) and sections (5 μm) were cut onto charged glass slides (Superfrost Plus Yellow) and rehydrated. Antigen retrieval was performed in citrate buffer pH 6.0 (sodium citrate 10 μM) at 90°C for 20 min. The sections were permeabilized (bovine serum albumin 10 mg/ml, horse serum 5%, Triton 0.5%, and sodium azide 0.02%) for 2 h and incubated with anti-CD8-FITC (BioLegend Cat# 100705, RRID:AB_312744), anti-CD103 (BioLegend Cat# 121426, RRID:AB_2563691), anti-PD-1-APC (BioLegend Cat# 135209, RRID:AB_2251944), anti-TIM-3-PE/Dazzle (BioLegend Cat# 134014, RRID:AB_2632738), anti-GZMB (BioLegend Cat# 372222, RRID:AB_2728389), anti-IFNγ-PE and anti-TCF-1 primary antibodies at room temperature (RT) for 18 h. The anti-CD103, anti-GZMB and anti-TCF-1/7 mAbs were revealed with a secondary antibody, either AF-647 (Jackson ImmunoResearch Labs Cat# 711-585-152, RRID:AB_2340621) or AF-594 (Jackson ImmunoResearch Labs Cat# 711-605-152, RRID:AB_2492288). The nuclei were counterstained with Hoechst (Invitrogen) for 10 min. The sections were mounted with Vectashield (Vector Laboratories).

### Human Samples of Melanoma and Skin Control

Twelve paraffin blocks from resection procedures of confirmed melanoma patients were obtained from the Pathology department of Hospital de Oncología Centro Médico Nacional Siglo XXI, and seven healthy control skin samples were obtained from a repository of biological tissues derived from non-cancerous surgical procedures. Both melanoma and skin samples were obtained with approval of our Institutional Scientific and Ethics Board of Reviews (protocol R-2019-785-05). After 2 years of clinical follow up, the melanoma patients were classified as “metastatic” when patients showed cancer spreading to other organs (*N* = 5) or “disease-free” when there was no tumor evidence after this period of time (*N* = 7) (**Supplementary Table 1**).

### Immunofluorescence Assays of Human Samples

Fifteen μm control skin or tumor sections were placed in charged glass slides (Superfrost Plus Green). Slides were placed into a stove (70°C) for 40 min in order to remove the paraffin excess. Tissues were rehydrated with a Xylene/ Ethanol train of solvents. Antigen retrieval was performed using citrate buffer pH 6.0 (sodium citrate 10 μM) at 120°C for 20 min. Skin samples were permeabilized for 3 h with a perm solution that contained 10mg/ml bovine serum albumin, 5% horse serum, 0.02% sodium azide, and 0.4% Triton. Following permeabilization the tissues were incubated with different primary antibodies: anti-CD8 (Bio-Rad Cat# MCA351G, RRID:AB_877504), anti-CD103 (LifeSpan, Cat# LS-C45284, RRID:AB_2296301), anti-PD-1 (Abcam, Cat# ab175391, RRID:AB_2868534), anti-CD69 (Abcam Cat# ab52587, RRID:AB_881954), or anti-TCF-1/7-AF-488 (Cell Signaling Technology Cat# 6444, RRID:AB_2797627) for 18 h. Incubation with secondary mAb AF-488 (Jackson ImmunoResearch Labs Cat# 711-545-152, RRID:AB_2313584), AF-594 (Jackson ImmunoResearch Labs Cat# 712-585-150, RRID:AB_2340688), and AF-647 (Jackson ImmunoResearch Labs Cat# 715-605-150, RRID:AB_2340862) was performed for 2 h. When specified, fluorescent conjugated antibody anti-IFN-γ-FITC (SONY BIOTECHNOLOGY, Cat# 3132520, RRID:AB_2868456) was incubated for 2 h. Nuclei were stained with Hoechst (Invitrogen) for 10 min. Sections were mounted with Vectashield (Vector Laboratories) and stored at 4°C. Quantification of positive CD8^+^ CD103^+^ CD69^+^ and CD8^+^ CD103^+^ TCF1^+^ cells was performed in highly infiltrated areas. A total of 100 cells were counted in each infiltrated area, registering positive cells. This process was repeated in three different tissue areas and the mean percentage of triple positive cells was obtained from each patient.

### Confocal Microscopy

Micrographs were obtained on a Nikon Ti Eclipse inverted confocal microscope (Nikon Corporation) using NIS Elements v.4.50. Imaging was performed using a 20x (dry, NA 0.8) objective lens. Zoom was performed at 3.4x and Digital Zoom was performed when specified. Images were analyzed using FIJI ImageJ Software (ImageJ software, National Institutes of Health; http://rsbweb.nih.gov/ij/).

### Statistics

Statistical analysis was performed using Prism 6.0 (GraphPad Software Inc., La Jolla, CA, USA). Statistical significance was calculated using unpaired two-tailed Student’s t-test for two groups, and one-way or two-way ANOVA with Tukey’s multiple comparison test for more than two groups. Statistical significance for tumor-free percentage was calculated using Log-rank (Mantel-Cox) test. Statistical significance was defined as * p < 0.05, ** p < 0.01, *** p < 0.001, **** p < 0.0001.

## Results

### Immunization With Porins Elicits Prophylactic and Therapeutic Melanoma Control

**Figure 1:**
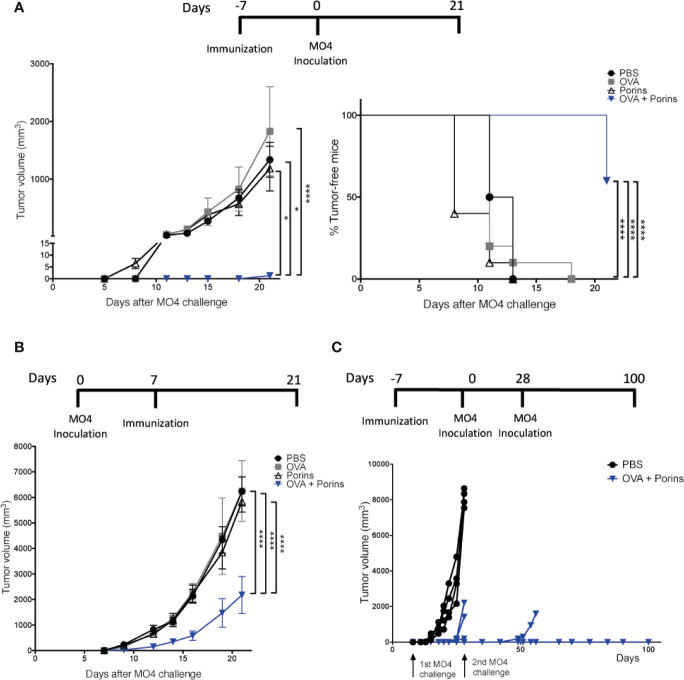
Porins as adjuvant induce an effective prophylactic and therapeutic anti-tumoral response. **(A)** C57BL/6 mice were immunized s.c. in the left back, with OVA + Porins or single inoculated with OVA, Porins or PBS, as control groups. Seven days later, 2.5 x 10^5^ MO4 cells were inoculated s.c. in the same place as the immunization. The tumor volume and the tumor-free mice were scored every 3 days starting on day 7 until day 21. **(B)** C57BL/6 mice were challenged with MO4 and 7 days later were immunized with OVA + Porins or inoculated with OVA, Porins or PBS. **(C)** Mice were immunized with either OVA + Porins (*N* = 7) or PBS and challenged with MO4 cells [as in **(A)**], and mice that were tumor-free by day 28 (*N* = 4) were re-challenged with a new dose of MO4 cells. Statistical data were pooled from three independent experiments with three mice per experimental condition. *p < 0.05, ****p < 0.0001.

We first determined whether the *S.* Typhi Porins could be used as a PAMP-adjuvant in a murine melanoma model using two different vaccination strategies exemplifying prophylactic and therapeutic approaches. Mice were immunized s.c. in the left flank with the model antigen OVA and the Porins as adjuvant. The same immunization conditions were used for the control groups: OVA-alone, Porins-alone and PBS. In the prophylactic approach, mice were challenged s.c. at the same place with the OVA-expressing B16-F10 melanoma cell line (MO4) 7 days post-immunization and the mice were followed for up to 21 days. The results showed that a prophylactic immunization with OVA + Porins powerfully suppressed tumor growth, with 60% of immunized mice clear of tumor and the rest bearing tumors under 2 mm^3^ in size (**Figure 1A**). Meanwhile all control mice had established large tumors.

Effective therapeutic responses are substantially more challenging, and we sought to assess whether the prophylactic protection of Porins was also capable to control already established tumors. For this, mice were first inoculated s.c. with MO4, and 7 days later immunized s.c. with the conditions mentioned above. Again, the combination of OVA + Porins was able to significantly control the tumor growth (**Figure 1B**). We then tested whether Porins could induce a protective immune memory. Since in the therapeutic experiment all OVA + Porins immunized mice developed small tumors, we evaluated memory in the prophylactic scheme, re-challenging tumor-free mice at day 28 with another MO4 inoculation. We observed that by the end of the 100 days following period, three out of four of the re-challenged mice were still tumor-free (**Figure 1C**). Altogether, these results support that the Porins from *S.* Typhi are an efficient prophylactic and therapeutic PAMP-adjuvant, also inducing a long-lasting protective memory against melanoma.

### Porins Elicit Circulatory and Tissue-Resident Memory T Cells

**Figure 2:**
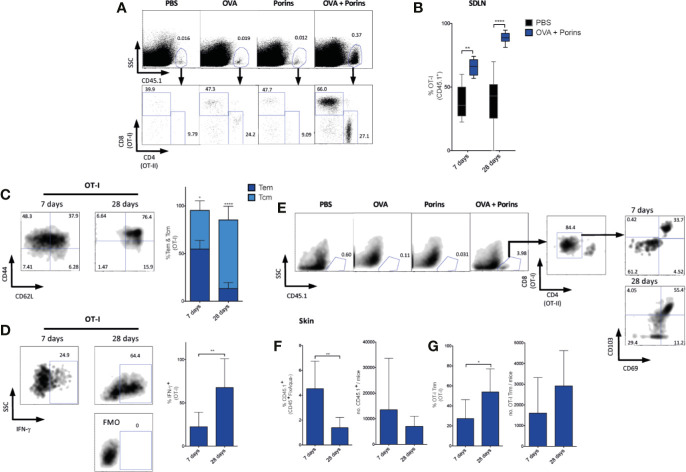
Porins induce circulating and tissue-resident memory T cells. C57BL/6 mice were i.v. transferred with OVA-specific CD8^+^ CD45.1^+^ T cells (OT-I) and CD4^+^ CD45.1^+^ T cells (OT-II) and a day later, i.d. immunized in both ears with OVA + Porins, and OVA, Porins or PBS as controls. Generation of the following populations was analyzed by flow cytometry in skin-draining lymph nodes (SDLN) **(A–D)** and skin **(E–G)** 7 and 28 days post-immunization as marked in the specific figure. **(A)** OVA-specific CD45.1^+^, OT-I and OT-II cells. **(B)** Comparison of SDLN OT-I frequencies induced by the immunization and PBS groups. **(C)** OT-I Tcm (CD44^+^ CD62L^+^) and OT-I Tem (CD44^+^ CD62L^-^). **(D)** IFN-γ positive OT-I cells from SDLN cells re-stimulated with OVA peptides (SIINKFEL and 323-339). **(E)** OT-I Trm T cells (CD103^+^ CD69^+^), with percentage and absolute numbers of CD45.1^+^ **(F)** and OT-I Trm T cells **(G).** Statistical data were pooled from three independent experiments with 3 mice per experimental condition. *p < 0.05, **p < 0.01, ****p < 0.0001.

To better understand the adjuvant mechanism of Porins, we evaluated its capacity to generate T cell memory subsets that are essential for tumor control. For this, we tracked the immune response of OVA-specific T cells using the well-studied adoptive transfer model of OT-I and OT-II CD45.1^+^ T cells, which express transgenic TCRs specific for the CD8 and CD4 OVA epitopes, respectively, in CD45.2 mice that were immunized with OVA + Porins 1 day after the adoptive transfer. We observed a 25-fold expansion of CD45.1^+^ cells in the OVA + Porins group by day 7 that consisted of both OT-I and OT-II T cells (**Figure 2A**). The OT-I frequency in skin-draining lymph nodes (SDLN) at day 7 and 28 was significantly higher for OVA + Porins compared with PBS (**Figure 2B**), while OT-II cells were only significantly increased at day 7 (**Supplementary Figure 1A**). When we evaluated the two main types of circulating memory in SDLN, we observed Tcm (CD44^+^ CD62L^+^) and Tem (CD44^+^ CD62L^-^) cells only in the OVA + Porins immunized mice, both types by day 7, and mostly Tcm cells by day 28 (**Figure 2C**). Both OT-I (**Figure 2C**) and OT-II (**Supplementary Figure 1B**) observed a similar pattern of memory formation. No memory cells were observed in the control groups (data not shown). We also tested the induced T cells to produce IFN-γ by re-stimulating them ex vivo with CD8 and CD4 specific OVA peptides. IFN-γ−producing cells comprised 25% and 60% of OT-I T cells by days 7 and 28, respectively (**Figure 2D**). Likewise, we observed IFN-γ^+^ OT-II T cells (**Supplementary Figure 1C**), although it was significantly lower than the OT-I response, particularly by day 28 (**Supplementary Figure 1D**).

Since Porins facilitate formation and expansion of circulating memory cells in the SDLN, we also evaluated its capacity to induce Trm T cells (CD103^+^ CD69^+^) in the skin, perhaps the most relevant population for melanoma control. We used the same adoptive transfer and immunization strategy explained above, again observing that only the OVA + Porins mice harbored CD45.1^+^ cells in the skin 7 and 28 days after immunization (**Figures 2E, F**). Both OT-I and OT-II T cells were able to form Trm T cells (**Figures 2E, G**; **Supplementary Figure 1E**), but again with significant reduced percentage for OT-II cells (**Supplementary Figure 1F**). Thus, Porins reinforce the formation of IFN-γ-producing T cells and a pool of early and lasting Tcm and Tem CD8^+^ T cells at draining lymph nodes and Trm CD8^+^ T cells at the site of immunization.

### Formation of Functional Trm T Cells Correlates With Melanoma Protection

**Figure 3:**
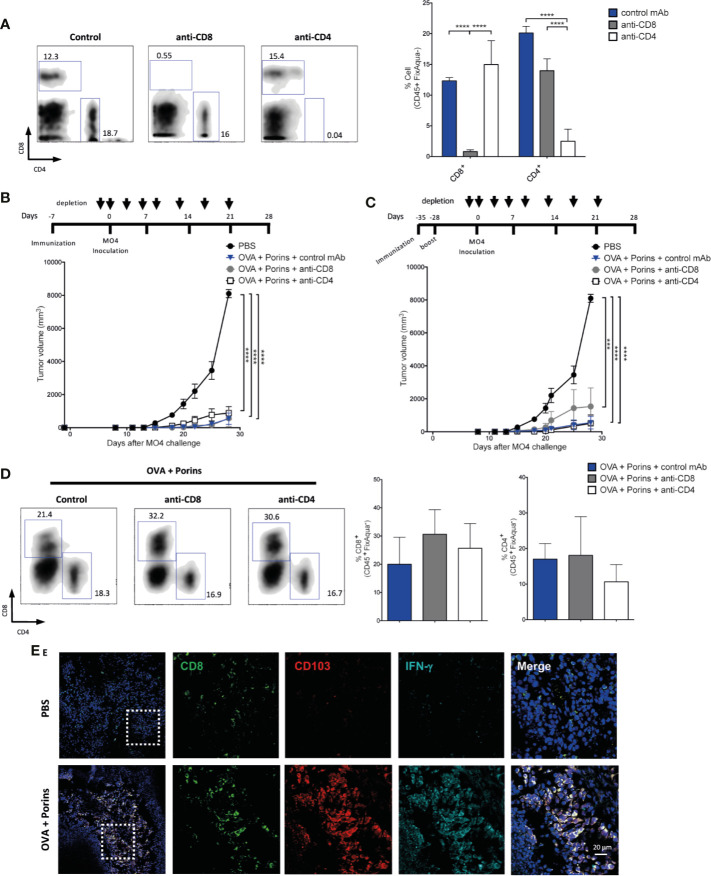
Porins induce functional tumor-infiltrating Trm T cells that correlate with tumor growth control. Immunized mice were depleted of CD8^+^ and CD4^+^ T cells **(A)**, and tumor control was assessed after two different immunization schemes, 7 days before MO4 inoculation **(B)** or 35 days before with boosting at day -28 **(C)**. Arrows indicate the anti-CD8 and anti-CD4 mAb injection days. **(D)** Flow cytometry charts and plots of the frequency of CD8^+^ and CD4^+^ TILs. **(E)** CD103^+^ (red) CD8^+^ (green) T cells expressing IFN-γ (cyan) from tumor of the prime/boost immunization scheme (representative immunofluorescence micrographs from a zoomed area) (Scale bar = 20μm). Statistical data were pooled from three independent experiments with 3–4 mice per experimental condition. ***p < 0.001, ****p < 0.0001.

To address the individual contributions of different memory T cell populations for protection against melanoma growth, we eliminated the circulating CD8^+^ or CD4^+^ T cells injecting i.p. eight doses of anti-CD8 or anti-CD4 mAbs. For this, we tested two immunization schemes: 1) 7 days before the MO4 melanoma cell challenge, and 2) 35 days before challenge with one boost at day -28. Similar experimental approaches have previously documented that skin and tumor-infiltrating Trm T cells are resistant to antibody-dependent depletion (38, 41). The efficiency of circulating T cell depletion is shown in **Figure 3A** and the immunization and T cell depletion schemes in **Figures 3B, C** (top). We observed tumor growth control in spite of depletion of circulating CD8^+^ or CD4^+^ T cells (**Figures 3B, C**, bottom), seen also by the percentage of tumor-free mice (**Supplementary Figures 2A, B**). Indeed, we corroborated that tumor-infiltrating CD8^+^ and CD4^+^ T cells were not affected by depletion of circulating T cells in the OVA + Porins-immunized mice 21 days post MO4 inoculation (**Figure 3D**). Worth mentioning, an immunization scheme 28 days before challenge and with no additional boost was not able to control the tumor growth in the absence of the circulating T cells (**Supplementary Figure 2C**). In order to evaluate Trm formation in the tumor stroma after a prime/boost immunization we evaluate the presence and function of Trm T cells by immunofluorescence (IF) microscopy. As it can be observed in **Figure 3E**, there is an increased infiltration of CD8^+^ T cells that express CD103 in the immunized mice compared with control mice. Importantly, the Trm T cell population in the tumor stroma express IFN-γ (**Figure 3E**) and granzyme B (GZMB) (**Supplementary Figure 3A**). The Trm formation in the tumors is also not affected by depletion of circulating T cells (**Supplementary Figure 3B**). Taken together, these data support that Porins strongly shape a tumor stroma Trm T cell milieu associated with the control of tumor growth, that can be explained by an early seeding of functional Trm T cells that need to be reinforced to be protective at late time points.

**Figure 4:**
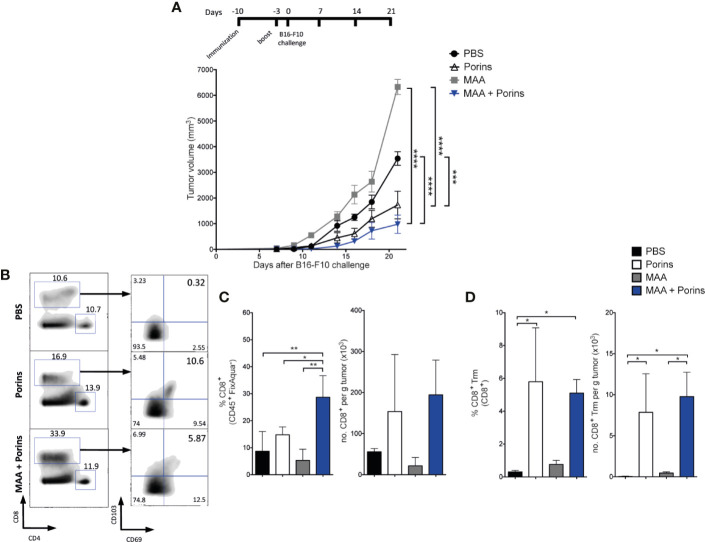
Porins induce protection against native melanoma antigens. **(A)** Mice were immunized s.c. with Porins, TRP-2 and gp100, two melanoma-associated antigens (MAA), 10 and 3 days before B16-F10 challenge and the control groups: Porins-alone, MAA-alone and PBS. **(B)** Flow cytometry analysis of CD8^+^ TILs assessing the Trm phenotype 21 days after melanoma challenge. Frequencies and absolute numbers per tumor mass are given of CD8^+^ T cells **(C)** and CD8^+^ Trm T cells **(D)**. Statistical data were pooled from two independent experiments with 5 mice per experimental condition. *p < 0.05, **p < 0.01, ***p < 0.001, ****p < 0.0001.

To assess the extent of the Porins-induced melanoma protective T cell populations, we tested the Porins adjuvant capacity against two melanoma-associated antigens (MAA), TRP-2 and gp100, previously reported as critical antigens in cancer vaccine development (42). For this, mice were immunized at day -10 before challenge with the B16-F10 melanoma cell line, according to previously published immunization strategies (39, 40). A boost was executed 7 days after the first immunization, when a Trm population is already established (**Figure 2E**). We observed better control of melanoma in mice immunized with MAA + Porins than MAA-alone or PBS (**Figure 4A**). Here, Porins-alone with the boosting scheme showed efficient protection and no clear differences were found with MAA + Porins, suggesting the induction of protective responses by the exogenous antigens but also by tumor endogenous melanoma antigens. Considering that the tumor growth control is independent of circulating T cells and seems to be dependent of T cells seeded in the tumor stroma (**Figure 3**), we next evaluated the presence of TILs in the immunized mice. Even though in the MAA + Porins group a higher percentage of CD8^+^ TILs was observed, the tumor-density of total CD8^+^ T cells was similar among all groups (**Figures 4B, C**). We did not observe differences among the groups in the total CD4^+^ T cells (**Supplementary Figures 4A, B**). Importantly, we observed the CD8^+^ Trm T cell formation only in the MAA + Porins immunization and the Porins-alone, which are the groups that could control the tumor growth (**Figures 4A, D**), this was similar with the CD4^+^ Trm TILs (**Supplementary Figures 4A, C**) although its frequency was significantly lower (**Supplementary Figure 4D**). These results illustrate the extent of the use of the Porins as an adjuvant of tumor associated antigens as well as the high correlation of Trm T cells formation with the tumor growth control.

### Porins Induce Enhanced Adjuvant Protection More Than the Cholera Toxin B Subunit

**Figure 5:**
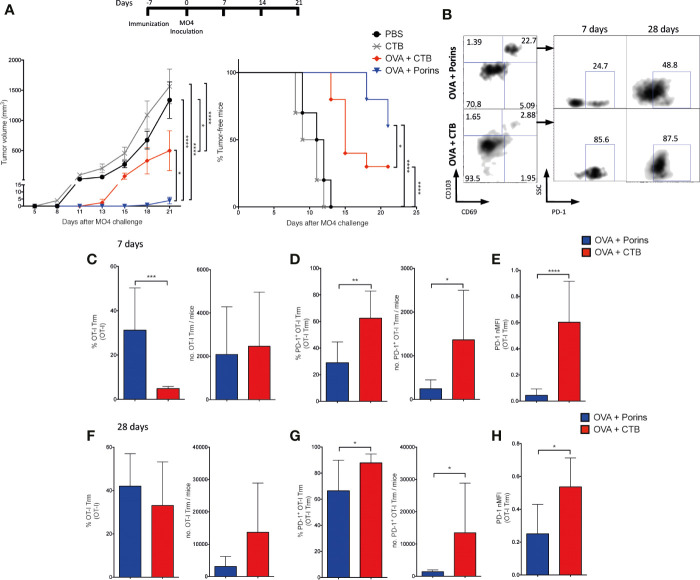
Porins induce better adjuvant protection than the B subunit of the Cholera toxin. **(A)** Mice were immunized subcutaneously with OVA + Porins or OVA + CTB and controls, challenged with MO4 cells and tumor volume and tumor-free mice were scored. Mice were adoptively transferred and immunized as in **Figure 2**, comparing the OVA + Porins against OVA + CTB immunization. **(B)** OT-I Trm T cells and PD-1^+^ cells at day 7 and 28. Percentage and absolute numbers of OT-I Trm T cells **(C)**, OT-I Trm T cells PD-1 positive **(D)** and the PD-1 normalized MFI from OT-I Trm T cells **(E)** at day 7; and the same analysis at day 28 **(F–H)**. Statistical data were pooled from three independent experiments with 3–4 mice per experimental condition. *p < 0.05, **p < 0.01, ***p < 0.001, ****p < 0.0001.

To put in context the anti-tumor adjuvant capacity of Porins (classical PAMP), we compared it against the CTB (non-classical PAMP), an adjuvant that also promotes the formation of protecting Trm T cells against melanoma (28). Mice were again immunized with each adjuvant and inoculated with MO4 melanoma cells 7 days later (**Figure 5A**, top). We observed that Porins and CTB exhibit prophylactic protection delaying tumor growth compared with control groups. However, there was a significantly better protection with OVA + Porins than with any other treatment, measured as tumor volume and percentage of tumor free mice (**Figure 5A**, left and right graphs, respectively). To gain insights into the relevance of the induced memory T cell populations, we compared the capacity of both CTB and Porins to generate CD8^+^ Trm T cells anticipating to observed more in the OVA + Porins mice correlating with the better protection. We also looked for early signals of exhaustion in this skin-infiltrating population by assessing PD-1 expression. For this, CD45.1^+^ OT-I and OT-II cells were tracked after adoptive transfer experiments similar to the ones for **Figures 2A–C**. We observed a greater expansion of skin-infiltrating CD45.1^+^ cells in OVA + CTB than in OVA + Porins mice at day 7 and 28 (**Supplementary Figures 5A–C**). Still, we observed a higher efficiency of formation of OT-I Trm in the OVA + Porins immunization group compared with the OVA + CTB (**Figures 5B, C**), but that was not reflected in absolute numbers because of the greatest expansion of CD45.1^+^ cells in OVA + CTB mice (**Supplementary Figure 5B**). Remarkably, we found that the OVA + Porins-induced OT-I Trm have a lower percentage and number of PD-1 positive cells (**Figure 5D**), also observed with a lower MFI (**Figure 5E**). Similar data were obtained when cells were harvested 28 days post immunization (**Figures 5F–H**), suggesting that the enhanced protection conferred by OVA + Porins may be due to the low numbers of CD8^+^ Trm PD-1^+^ T cells. Somehow different, the OT-II Trm T cell frequency was greater for OVA + Porins than OVA + CTB, but induced-OT-II Trm T cells with both adjuvants have similar percentages of PD-1 positive cells at day 7 (**Supplementary Figure 5D**), which became more abundant in the OVA + Porins by day 28 (**Supplementary Figure 5E**). In summary, even though CTB can promote a superior expansion of OVA-specific T cells in the skin, Porins are capable to promote a greater and more stable population of CD8^+^ Trm T cells with lower PD-1 expression as an early exhaustion signature, and perhaps because of that, Porins promoted a more efficient control of melanoma.

### Porins-Induced Progenitor Exhausted Trm T Cell Population Correlates With an Enhanced Protection

**Figure 6:**
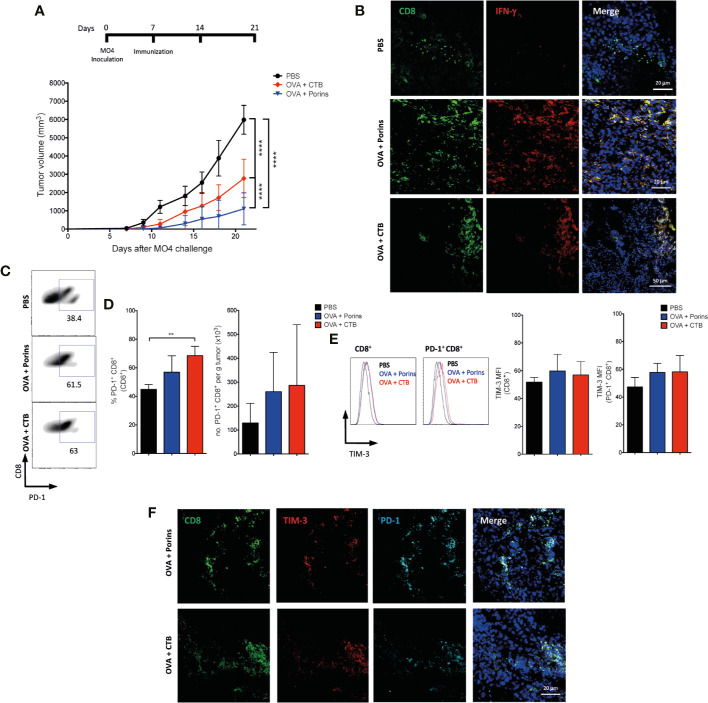
Porins induce functional TIM-3^+^ PD-1^+^ CD8^+^ T cells in a therapeutic scheme. **(A)** Mice were inoculated with MO4 cells and immunized s.c. with OVA + Porin, OVA + CTB or PBS control, data is presented 21 days after the melanoma challenge. **(B)** CD8^+^ (green) T cells expressing IFN-γ (red) from tumors (representative immunofluorescence micrographs from a zoomed area). **(C)** Flow cytometry of CD8^+^ PD-1^+^ TILs and **(D)** their percentages. **(E)** TIM-3 medium fluorescent intensity (MFI) from CD8^+^ and CD8^+^ PD-1^+^ TILs. **(F)** CD8^+^ (green) T cells expressing TIM-3 (red) and PD-1 (cyan) from tumors (representative immunofluorescence micrographs from a zoomed area) (Scale bar = 20μm). Statistical data were pooled from three independent experiments with three mice per experimental condition. **p < 0.01, ****p < 0.0001.

Since CTB and Porins immunization provides a suitable model to compare induced populations with protection, we compared the capacity of both adjuvants to induced native T cell populations without the constraint of a T cell adoptive transfer (28). For this, we first compared the therapeutic protection rendered by OVA + CTB and OVA + Porins in MO4 melanoma, observing again a more proficient protection with the latter (**Figure 6A**). Next, we assessed whether Porins were also able to induce functional T cells evaluating the formation of IFN-γ-producing T cells. Indeed, we observed IFN-γ-producing CD8^+^ TILs in both immunizations, but lacking in the PBS control (**Figure 6B**). Flow cytometry analysis of CD8^+^ TILs showed a greater frequency of PD-1^+^ cells in OVA + CTB immunization that is not reflected in absolute numbers (**Figures 6C, D**). When we assessed the presence of potential terminally exhausted T cells, we were able to observe CD8^+^ TILs co-expressing PD-1 and TIM-3 in both immunizations with no difference in TIM-3 expression (**Figures 6E, F**). Importantly, we confirmed the functionality of CD8^+^ TIM-3^+^ TILs through expression of IFN-γ and GZMB, although there were no differences between both immunizations (**Supplementary Figures 6A, B**). IFN-γ secretion has previously been associated with terminally exhausted T cells (9, 10). Thus, this potentially terminally exhausted phenotype was also evaluated in mice immunized with MAA + Porins and with Porins-alone, observing in CD8^+^ and in CD8^+^ Trm TILs the co-expression of TIM-3 and PD-1 exclusively for the MAA + Porins immunization condition (**Supplementary Figures 7A, B**), these phenotypes have already been described in previous studies (43, 44). Nevertheless, the presence of TIM-3^+^ CD8^+^ Trm TILs did not correlate with tumor growth control (see **Figure 4A** and **Supplementary Figure 7B**).

**Figure 7:**
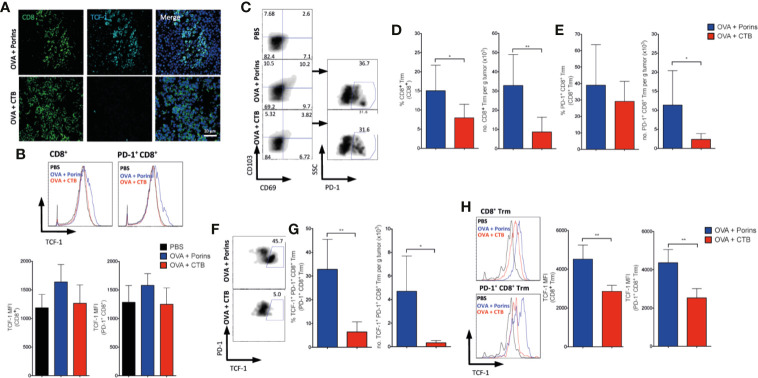
Porins-induced tumor-infiltrating TCF-1^+^ Trm CD8^+^ T cell correlates with melanoma protection. Vaccination scheme as in **Figure 6A**, data is presented 21 days after the melanoma challenge. **(A)** CD8^+^ (green) T cells expressing TCF-1 (cyan) from tumors (representative immunofluorescence micrographs from a zoomed area) (Scale bar = 20μm). **(B)** TCF-1 medium fluorescent intensity (MFI) from CD8^+^ and PD-1^+^ CD8^+^ TILs. **(C)** Flow cytometry of PD-1^+^ expressing CD8^+^ Trm T cells. Frequencies and absolute numbers of CD8^+^ Trm **(D)** and PD-1^+^ CD8^+^ Trm **(E)**. **(F)** Flow cytometry of CD8^+^ Trm T cells expressing PD-1^+^ and TCF-1^+^. **(G)** Frequencies and absolute numbers of TCF-1^+^ PD-1^+^ CD8^+^ Trm T cells. **(H)** TCF-1 MFI from CD8^+^ Trm and PD-1^+^ CD8^+^ Trm. Statistical data were pooled from three independent experiments with 3–4 mice per experimental condition. *p < 0.05, **p < 0.01.

Considering that the TIM-3^+^ PD-1^+^ CD8^+^ TILs induced by Porins did not show correlation with protection, we evaluated the tumor-infiltrating CD8^+^ T cells with a TCF-1^+^ progenitor exhausted phenotype. Immunofluorescence staining of the tumor stroma showed CD8^+^ TCF-1^+^ cells, solely in the OVA + Porins immunization (**Figure 7A**). An increase in the TCF-1 MFI in the CD8^+^ and CD8^+^ PD-1^+^ TILs was also observed by flow cytometry in the OVA + Porins condition, but did not reach a statistically difference compared with OVA + CTB (**Figure 7B**). Considering the higher TCF-1 expression of the OVA + Porins-induced CD8^+^ TILs, we evaluated if the progenitor exhausted phenotype was derived from a Trm population. We observed a greater infiltrate of CD8^+^ Trm T cells in OVA + Porins-immunized mice than in OVA + CTB (**Figures 7C, D**). The same was observed with the percentage and tumor-infiltrate density of the PD-1^+^ CD8^+^ Trm T cells (**Figures 7C, E**). Interestingly, among tumor-infiltrating cells the population that expressed TCF-1 corresponded to the PD-1^+^ CD8^+^ Trm T cells, only noticed in the tumors from OVA + Porins-immunized mice (**Figures 7F, G**; **Supplementary Figure 8**), which is also supported by the greater TCF-1 MFI of the CD8^+^ Trm T cells and the PD-1^+^ subset (**Figure 7H**). These results indicate that Porins induce a progenitor exhausted Trm T cell population that correlates with tumor growth control.

### Porins-Induced Progenitor Exhausted Trm T Cell Population Correlates With Anti-PD-1 Immunotherapy Cooperation

**Figure 8:**
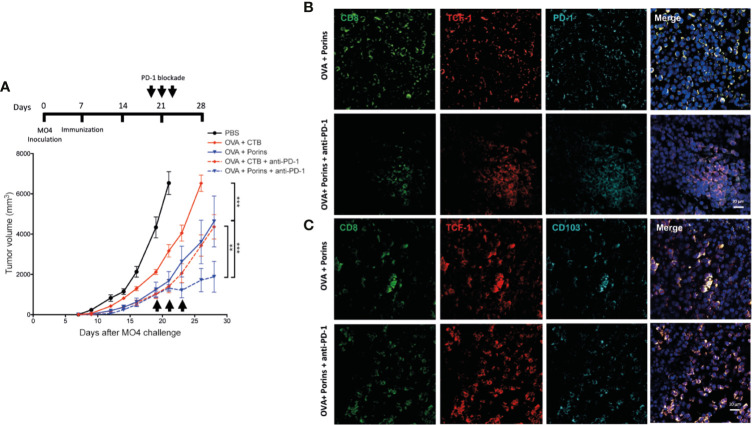
Porins-induced progenitor exhausted Trm CD8^+^ T cells correlate with enhanced response to anti-PD-1 immunotherapy. **(A)** Mice immunized and challenged as in **Figure 6A** were treated with anti-PD-1 immunotherapy (arrows), and tumors were harvested at day 28. Analysis of TCF-1^+^ (red) CD8^+^ (green) T cells expressing PD-1 (cyan) **(B)** or CD103 (cyan) **(C)** from the tumors of OVA + Porins-immunized mice with or without anti-PD-1 mAb, representative immunofluorescence micrographs from a zoomed area (Scale bar = 20μm). Statistical data were pooled from three independent experiments with 3-4 mice per experimental condition. **p < 0.01, ***p < 0.001.

Because the tumor-infiltrating progenitor exhausted TCF-1^+^ PD-1^+^ CD8^+^ T cells are documented to be the population that responds to the anti-PD-1 immunotherapy (9, 17), we administrated three doses of anti-PD-1 mAb at days 19, 21, and 23 in mice therapeutically immunized with either OVA + CTB or OVA + Porins (**Figure 8A**, top) (9). For this experiment, the anti-PD-1 immunotherapy was carried out only in mice harboring tumors of similar size from both immunization protocols to level the starting point of tumor growth, in which we were going to evaluate the response to immunotherapy. We observed that while progression of tumors in OVA + CTB mice was not altered by the anti-PD-1 mAb, immunotherapy halted further tumor growth in the OVA + Porins mice (**Figure 8A**, bottom). These data support that Porins promote the formation of a CD8^+^ Trm T cell population with a progenitor exhausted phenotype and function. A clear difference between the immunization conditions tested was the greater capacity of Porins to generate a PD-1-expressing CD8^+^ Trm T cells that also express TCF-1 (**Figures 7F, G**; **Supplementary Figure 8**). Thus, we also evaluated the formation of the TCF-1^+^ populations in the mice immunized with Porins treated or not with anti-PD-1 mAb. We observed the formation of TCF1^+^ PD-1^+^ CD8^+^ T cells clusters (**Figure 8B**), as well as an increase in the TCF-1^+^ CD103^+^ CD8^+^ T cells (**Figure 8C**) in the tumor stroma of mice treated with Porins and anti-PD-1 mAb, suggesting the expansion of this population after the immunotherapy. In contrast the frequency of TIM-3^+^ PD1^+^ CD8^+^ T cell population after the PD-1 blockade was similar that in untreated Porin-immunized mice (**Supplementary Figure 9**). Altogether these results argue for an important role of the progenitor exhausted Trm T cell population for control of melanoma growth, independently or in cooperation with anti-PD-1 immunotherapy.

### Progenitor Exhausted Trm T Cells in the Tumor Stroma of Melanoma Patients Also Correlate With Disease Control

**Figure 9:**
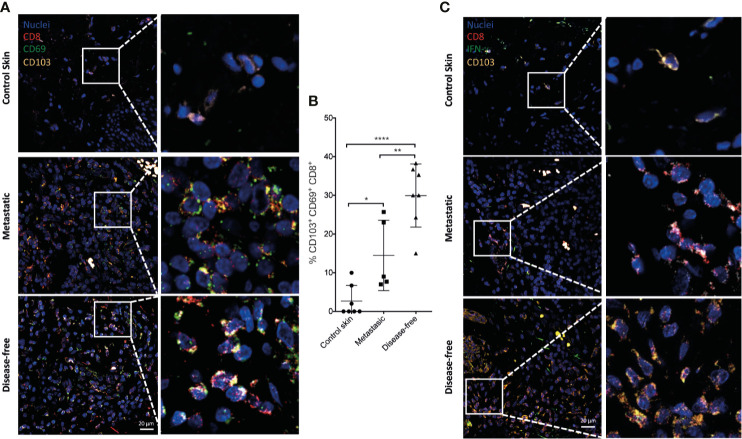
IFN-γ-producing Trm CD8^+^ TILs correlate with disease control in melanoma patients. Merged images of CD103^+^ (yellow) CD69^+^ (green) CD8^+^ (red) **(A)** and CD103^+^ (yellow) IFN-γ^+^ (green) CD8^+^ (red) **(C)** TILs from healthy control skin or from melanoma derived from metastatic or disease-free patients (representative immunofluorescence micrographs from a zoomed area) (Scale bar = 20 μm). **(B)** Percentages of CD103^+^ CD69^+^ CD8^+^ T cells are shown. Statistical data were pooled from a comparison of healthy skin (*N* = 7), and metastatic (*N* = 5) and disease-free melanoma patients (N = 7). *p < 0.05, **p < 0.01, ****p < 0.0001.

**Figure 10:**
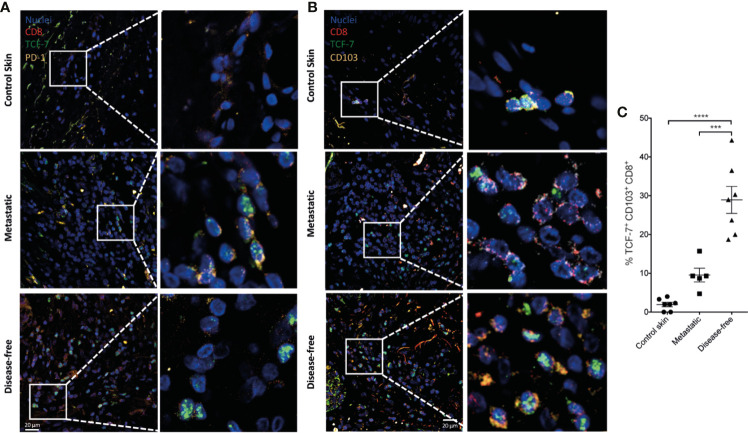
TCF-7^+^ progenitor exhausted Trm CD8^+^ TILs correlates with disease control in melanoma patients. Merged images of TCF-7^+^ (green) PD-1^+^ (yellow) CD8^+^ (red) **(A)** and TCF-1^+^ (green) CD103^+^ (yellow) CD8^+^ (red) **(B)** TILs from healthy control skin or from tumor stroma derived from metastatic or disease-free melanoma patients (representative immunofluorescence micrographs from a zoomed area) (Scale bar = 20μm). **(C)** Percentages of TCF-7^+^ CD103^+^ CD8^+^ T cells are shown. Statistical data were pooled from a comparison of healthy skin (*N* = 7), and metastatic (*N* = 5) and disease-free melanoma patients (*N* = 7). ***p < 0.001, ****p < 0.0001.

We determined whether the Trm T cell populations observed after Porin immunization could also be present in human melanoma tumors. For this, we assessed resection products of five metastatic and seven disease-free melanoma patients after a 2 years follow-up (**Supplementary Table 1**). We observed an increase of CD103^+^ CD69^+^ CD8^+^ T cells in the tumor stroma of disease-free patients compared with metastatic patients and healthy control skin (CS) (**Figures 9A, B**; **Supplementary Figure 10A**). Importantly, the Trm CD8^+^ TILs of melanoma patients expressed IFN-γ; which were increased in disease-free patients compared with metastatic patients (**Figure 9C**; **Supplementary Figure 10B**). We also evaluated the presence of progenitor exhausted T cells. As shown in **Figure 10A**, there was an increment of TCF-7^+^ PD-1^+^ CD8^+^ T cells in melanoma patients compared with CS (**Figure 10A**; **Supplementary Figure 11A**). Remarkably, there is also a significant increment of CD103^+^ CD8^+^ TILs that express TCF-7 in disease-free patients compared with CS and metastatic patients (**Figures 10B, C**; **Supplementary Figure 11B**). Altogether, these results indicate that the populations induced by Porins immunization are present in human melanoma, in which they also seem to correlate with disease control, supporting the findings observed in the B16-F10 melanoma model and indicating the relevance of the induction or expansion of this population by cancer vaccination strategies.

## Discussion

A CD8^+^ T cell signature has traditionally marked good prognosis in many types of cancers (45), but it is only more recently that we are beginning to appreciate the great complexity of T cell subpopulations inhabiting the tumor stroma and the infiltrating lymph nodes. Successful immune-based therapies should expand and rescue effector functions of those populations with the most anti-tumor activity. We showed here that *S.* Typhi Porins are an effective adjuvant conferring prophylactic and therapeutic protection against melanoma bearing a model antigen (OVA) or native MAAs (TRP-2/gp100) indicating the large spectrum of protection and that includes endogenous antigens. In agreement, Porins were able to induce IFN-γ-producing T cells, Tcm and Tem cells in SDLN as well as the formation of Trm T cells in the skin and in the melanoma stroma.

We observed an early induction of Trm T cells that correlated with tumor protection (37, 38), contrary to studies in which Trm maturation took at least 4 weeks (46). Other authors support circulating CD4^+^ and CD8^+^ T cell memory formation as early as 3–5 days after antigen encounter (47–49). We were also able to induce a lasting Trm population, but to achieve an effective anti-tumoral response independently of circulating memory T cells (37, 38), it required a prime/boost scheme most likely to sustain the numbers of Trm cells required for protection (28, 43, 50, 51). Furthermore, we also observed a long-lasting protective memory when we re-challenged tumor free mice, which remained healthy for additional 70 days. Therefore, protection is most likely explained by the expansion of antigen-specific memory T cells in the lymph nodes, which develop into functional Trm T cells seeded in the tumor stroma. Although we did not formally prove that protection is dependent on CD4^+^ and CD8^+^ Trm, we have previously reported that protection using the B16-F10 melanoma model is highly dependent on CD8^+^ T cells, and in a lesser extent also on CD4^+^ T cells (52). Here, tumor protection was still observed after depletion of circulating T cells but in the presence of functional Trm T cells, suggesting that CD4^+^ and CD8^+^ Trm T cells were responsible for protection. Although Tem cells can be present in the tumor stroma, these cells still maintain the potential to recirculate (28, 37). Previous reports also support that Trm T cells are highly relevant for tumor control (4, 5, 37, 43). However it is possible that in the absence of sustain functional Trm, other memory T cell populations could be relevant in tumor protection (37, 38). Supporting the findings in the B16-F10 murine model, we present evidence of the presence of functional IFN-γ-producing Trm T cells in melanoma patients, which are also associated with disease control. This is consistent with previous work correlating infiltrating Trm T cells and survival in immunized melanoma patients (53).

The search for measures to foster cancer control, as with vaccination or immunotherapy, has propelled the hunt for biomolecules that condition T cell effector functions, the identity of the populations that express them, and for strategies to restore their positive responses. CD8^+^ populations co-expressing markers of exhaustion, memory, effector functions and stemness have been often observed in tumor infiltrates, conceptually framing the idea of an ontogenic flow of exhausted memory cells sustained at the base by those with progenitor potential. Sade-Feldman M et al., defined at least six subpopulations after single cell RNA sequencing of tumor CD8^+^ T cells from melanoma patients. T cell populations expressing TCF-7 were enriched in immunotherapy responding patients (17). TCF-7^+^ progenitor exhausted T cells also seem to be the best marker of good prognosis, mainly documented for melanoma, but also for kidney and lung cancer patients (9, 15–17). In addition, the equivalent TCF-1^+^ exhausted population in mice has the potential to generate a terminally exhausted TIM-3^+^ population with functional effector function (9, 10). We document here that Porins induce IFN-γ- and GZMB-producing TIM-3^+^ CD8^+^ T cells in the tumor stroma; however, we were unable to correlate the density of TIM-3^+^ cells with neither enhanced or reduced protection. In contrast, the importance of the TCF-1^+^ Trm T cell populations was highlighted in the comparison between Porins and CTB. Although both Porins and CTB induced CD8^+^ Trm T cells in the tumor stroma, Porins were highly efficient to form TCF-1^+^ CD8^+^ Trm, contrary to CTB. This difference may be at least in part explained by their mechanisms to activate DCs, since Porins induce a transient and CTB a sustained activation (28, 33).

Our findings are also supported by a previous study, in which tumor-infiltrating TCF-1^+^ PD-1^+^ CD8^+^ T cells were capable to control the tumor in response to vaccination (54). Recently, it was determined in mice that one of the TCF-1^+^ exhausted subsets also express CD69, a tissue-resident memory marker, which is in agreement with our data (14). We also identified a TCF-7^+^ CD103^+^ CD8^+^ progenitor exhausted T cell population in the tumor stroma of human melanoma, which seems to be the equivalent to the progenitor exhausted Trm identified in mice. To the best of our knowledge, this is the first evidence of a CD8^+^ Trm T cell with a progenitor exhausted phenotype. Furthermore, this human progenitor exhausted Trm T cell also correlated with disease control in melanoma patients. Also relevant is that this protective population can be induced or expanded by immunization strategies that result in better melanoma control, such as the one we tried here with Porins as a cancer vaccine adjuvant.

Although our data support the existence of CD8^+^ Trm T cells that express the progenitor marker TCF-1, it has been previously reported that in virus and tumor models CD8^+^ Trm T cells express Blimp-1 (54–56), a transcriptional repressor of the *Tcf7* gene that encodes TCF-1. The capacity of Blimp-1 to promote terminally exhausted T cells has been previously documented (16, 57). On the contrary, Blimp-1 has also been associated with stemness, pluripotency, efficient memory T cell response, and lineage decisions in hematopoietic stem cells and in more mature immune cells (55, 58, 59), supporting the co-existence of this transcription factor and TCF-1 in cells that maintain progenitor potential. In this scenario, the studies mentioned above did not evaluate TCF-1 protein expression, and perhaps Blimp-1 only modulates *Tcf7* expression to open the way to form downstream populations, which while maintaining progenitor competence also start turning on additional terminal effector functions (56). In future studies, it would be important to determine Blimp-1 expression in the progenitor exhausted Trm CD8^+^ T cells induced by cancer vaccines.

There are several immunization strategies that induce an effective anti-tumoral response, but only few have been documented to cooperate with anti-PD-1 immunotherapy (21–24). Moreover, the strategies for recovery of TCF-1^+^ exhausted T cell populations that explain the cooperative mechanisms with PD-1 blockade are only beginning to be elucidated (54). Porins promoted formation of TCF-1^+^ PD-1^+^ CD8^+^ Trm T cells, leading us to assess whether they could also respond to anti-PD-1 immunotherapy. Indeed, we observed that Porins seem to sensitize mice for better control of melanoma growth upon immunotherapy. Worth mentioning, we observed a positive response even though we carried the anti-PD-1 immunotherapy at later times than in most melanoma murine models, when the tumor had already reached a considerable size. These data further highlight the efficacy of the early generation and expansion of progenitor exhausted Trm T cells in the tumor stroma as central for melanoma control independently of anti-PD-1 immunotherapy, but also priming it for additional strategies looking to reinforce anti-tumoral responses. It is interesting how these progenitor exhausted T cells converge as an important population for cancer control in both immunization and immunotherapy strategies. Understanding how these T cell populations are formed and modulated will be decisive for strategies of cooperative therapies, particularly because of the high number of patients with tumors refractory to immunotherapy or that relapse after an initial positive response (3, 60, 61).

**Figure 11:**
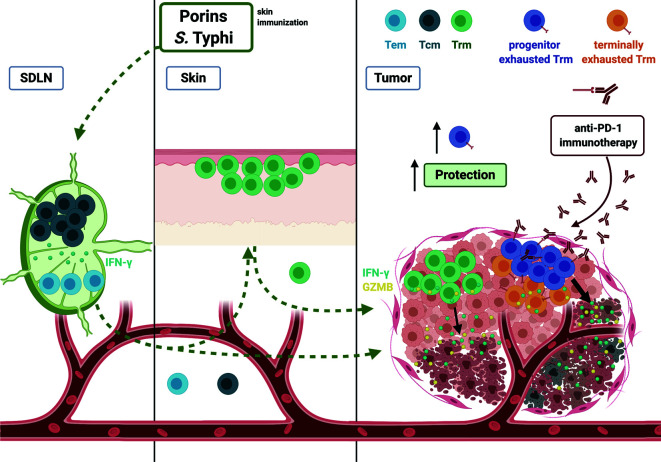
Model of vaccination strategy protecting against melanoma using *Salmonella* Typhi Porins. Depicted are the circulating from skin-draining lymph nodes (SDLN), skin and tumor-infiltrating Trm T cell response induced by Porin immunization. Porins as an adjuvant induce a circulating memory T cell response. In addition, the immunization induces a Trm response in the skin and a formation of functional Trm T cells in the tumor. A Porins immunization is capable to induce tumor-infiltrating TIM-3^+^ PD-1^+^ CD8^+^ Trm T cells (potential terminally exhausted Trm) and importantly TCF-1^+^ PD-1^+^ CD8^+^ Trm T cells (progenitor exhausted Trm), being the latter associated with tumor growth control and anti-PD-1 immunotherapy cooperation. Created with BioRender.com.

In summary, our data highlight the importance of adjuvants in cancer vaccines to sculpt the tumor microenvironment with the appropriate tumor-fighting populations, placing Porins as an adjuvant with the capacity to seed and expand CD8^+^ Trm T cells with a progenitor exhausted phenotype, which are very proficient to control melanoma growth and display responsiveness to anti-PD-1 immunotherapy (**Figure 11**). A combination of vaccination and immunotherapy strategies would help patients with aggressive cancers that are not benefited by traditional treatment schemes.

## Data Availability Statement

The raw data supporting the conclusions of this article will be made available by the authors, without undue reservation.

## Ethics Statement

The studies involving human participants were reviewed and approved by Institutional Scientific and Ethics Board of Reviews (protocol R-2019-785-051). Written informed consent for participation was not required for this study in accordance with the national legislation and the institutional requirements. The animal study was reviewed and approved by Institutional Ethics Committee and the Mexican national regulations on animal care and experimentation (R-2015-785-008).

## Author Contributions

RL-L performed the majority of the experiments, interpreted the data and drafted the manuscript. D-CM helped with the immunization experiments and tissue processing. O-BG helped with the tumor processing to obtain TILs. AT-S helped with the tissue processing and skin analysis. EH-M performed the IF of the mice tumors and CA-F and SD-R performed the IF of human melanoma tumors. AM selected the patients and performed the histological assays to identify the tumor infiltrating areas. E-FP discussed and revised the results and thoroughly revised the manuscript. CL-M produced Porins and conceived its use as adjuvants, helped with the design of the study and revised the manuscript. LB conceived, designed, directed the study and wrote the manuscript. All authors contributed to the article and approved the submitted version.

## Funding

RL-L is a doctoral student from the Programa de Doctorado en Ciencias Biomédicas, Universidad Nacional Autónoma de México (UNAM) and has received Consejo Nacional de Ciencia y Tecnología (CONACyT) fellowship 631989. This study was funded by CONACyT, grant number: SRE-CONACYT 263683 and SEP-CONACYT CB-2015-256402 (to CL-M), and principally by the Instituto Mexicano del Seguro Social (IMSS), R-2019-785-051 FIS/IMSS/PROT/PRIO/19/125 and R-2015-785-008 FIS/IMSS/PROT/G16/1606 (to LB).

## Conflict of Interest

CL-M is listed as inventor on a patent related to the use of Salmonella Porins as adjuvants and vaccines.

The remaining authors declare that the research was conducted in the absence of any commercial or financial relationships that could be construed as a potential conflict of interest.

## Abbreviations

CTB, nontoxic Cholera B subunit; GZMB, Granzyme B; MAA, melanoma associated antigen; Porins, highly purified outer membrane proteins; *S.* Typhi, *Salmonella* enterica serovar Typhi; Tcm, central memory T cell; Tem, effector memory T cell; TIL, tumor-infiltrating lymphocyte; Trm, tissue-resident memory.
