# Supplementary material for: Induction of Progenitor Exhausted Tissue-Resident Memory CD8+ T Cells Upon Salmonella Typhi Porins Adjuvant Immunization Correlates With Melanoma Control and Anti-PD-1 Immunotherapy Cooperation
Source: Front Immunol. 2020 Nov 6;11:583382. doi: 10.3389/fimmu.2020.583382 (PMC7682137; doi:10.3389/fimmu.2020.583382)
Supplement: Supplementary file 1 [file DataSheet_1.pdf]

Supplementary Material 1

Supplementary Table 1. Demographic data of melanoma patients.

PATIENTS	METASTATIC (M) / DISEASE-FREE (DF)	AGE	SEX	T	N	M	BRESLOW	CLINICAL EVOLUTION
465	DF	66	M	2	0	0	2 MM	DISEASE-FREE
91	DF	79	M	2	0	0	1.1 MM	DISEASE-FREE
8011	DF	73	F	4	1	0	4 MM	DISEASE-FREE
5017	DF	59	F	1	0	0	0.6 MM	DISEASE-FREE
6321	DF	72	M	1S	0	0	ND	DISEASE-FREE
2126	DF	53	F	1	0	0	0.8 MM	DISEASE-FREE
2030	DF	85	F	2	0	0	2 MM	DISEASE-FREE
5532	M	61	M	4B	1B	0	7 MM	LOCOREGIONAL PROGRESSION. PULMONARY, LIVER, MEDIASTINUM, ABDOMINOPELVIC METASTASIS
5837	M	85	M	1A	0	0	0.3 MM	LYMPH NODE METASTASIS
1628	M	67	F	4B	0	0	12 MM	PULMONARY AND LIVER METASTASIS
5835	M	74	F	3	0	0	3 MM	LYMPH NODE METASTASIS
7737	M	50	F	4B	1	0	11 MM	PULMONARY AND LIVER METASTASIS

TNM staging was done at the resection procedure in 2018 and the Clinical Evolution was updated after 2 years.

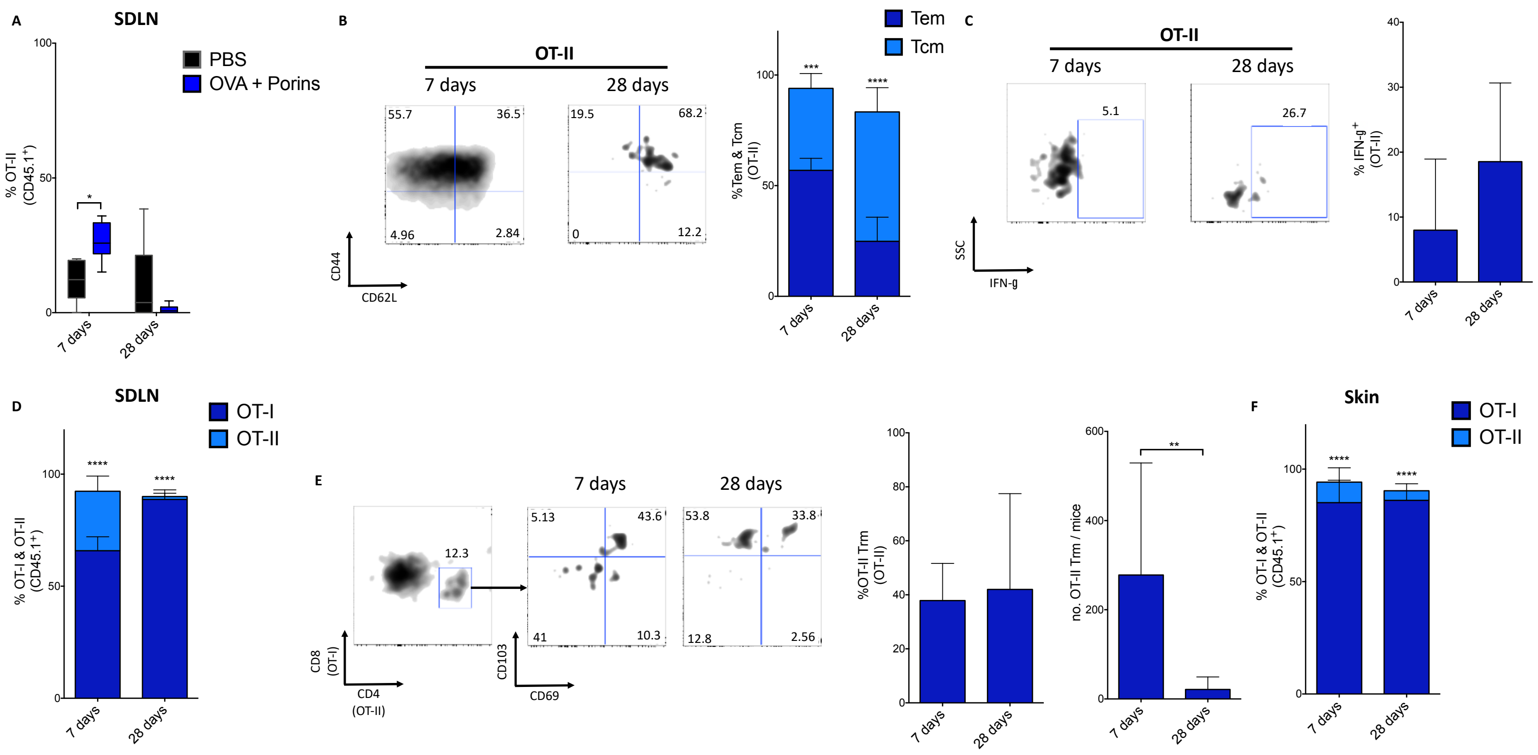

Supplementary Figure 1. OT-II circulating and tissue-resident memory cells. C57BL/6 mice were intravenously transferred with OVA-specific CD8^{+} CD45.1^{+} T cells (OT-I) and CD4 CD45.1^{+} T cells (OT-II) and a day later, intradermally immunized in both ears with OVA + Porins, and OVA, Porins or PBS as controls. **(A)** Skin-draining lymph nodes (SDLN) OT-II frequency induced by the immunization compared to the PBS group. Generation of the following populations was analyzed in SDLN **(B-D)** and of skin **(E-F)**, 7 and 28 days post-immunization as marked in the specific figure. **(B)** OT-II Tcm (CD62L^{+} CD44^{+}) and OT-II Tem (CD62L^{-} CD44^{+}). **(C)** IFN-γ positive OT-II cells from SDLN cells re-stimulated with OVA peptides (SIINKFEL and 323-339). **(D)** The percentage of OT-I and OT-II in SDLN. **(E)** OT-II Trm T cells (CD103^{+} CD69^{+}), with percentage and absolute numbers. **(F)** The percentage of OT-I and OT-II in the skin. Statistical data were pooled from three independent experiments with 3 mice per experimental condition. $^{**}p < 0.01$, $^{***}p < 0.001$, $^{****}p < 0.0001$.

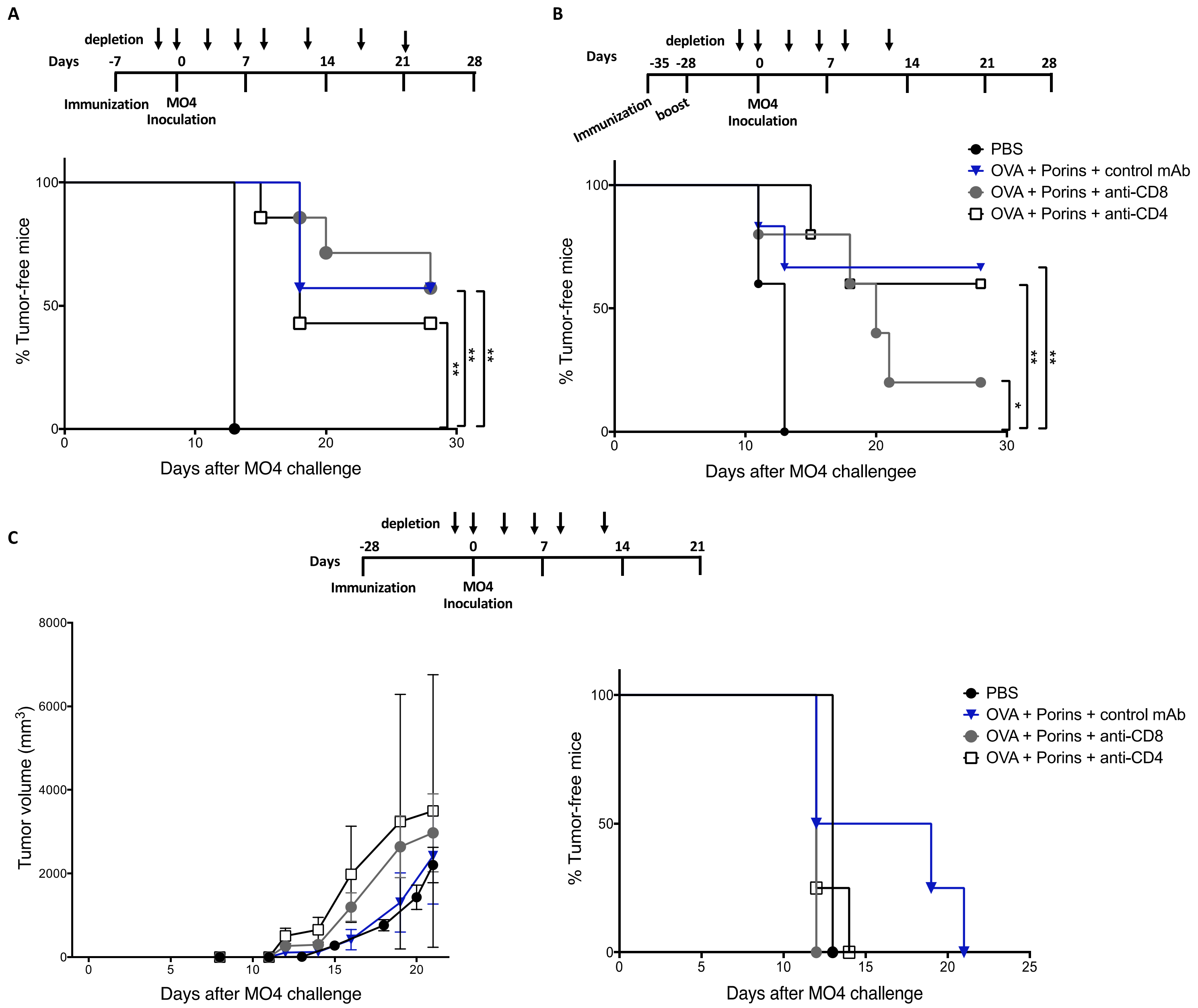

Supplementary Figure 2. Tumor growth with circulating lymphocyte elimination. Kinetics of tumor-free mice upon 7 days **(A)** and 35 days with boost **(B)** before MO4 inoculation, with arrows indicating the anti-CD8 and anti-CD4 mAb injection days. **(C)** Tumor growth and tumor-free mice upon 28 days before MO4 inoculation, with arrows indicating the anti-CD8 and anti-CD4 mAb injection days. Statistical data were pooled from one experiment with 5 mice per experimental condition. $^*p < 0.05$, $^{**} p < 0.01$.

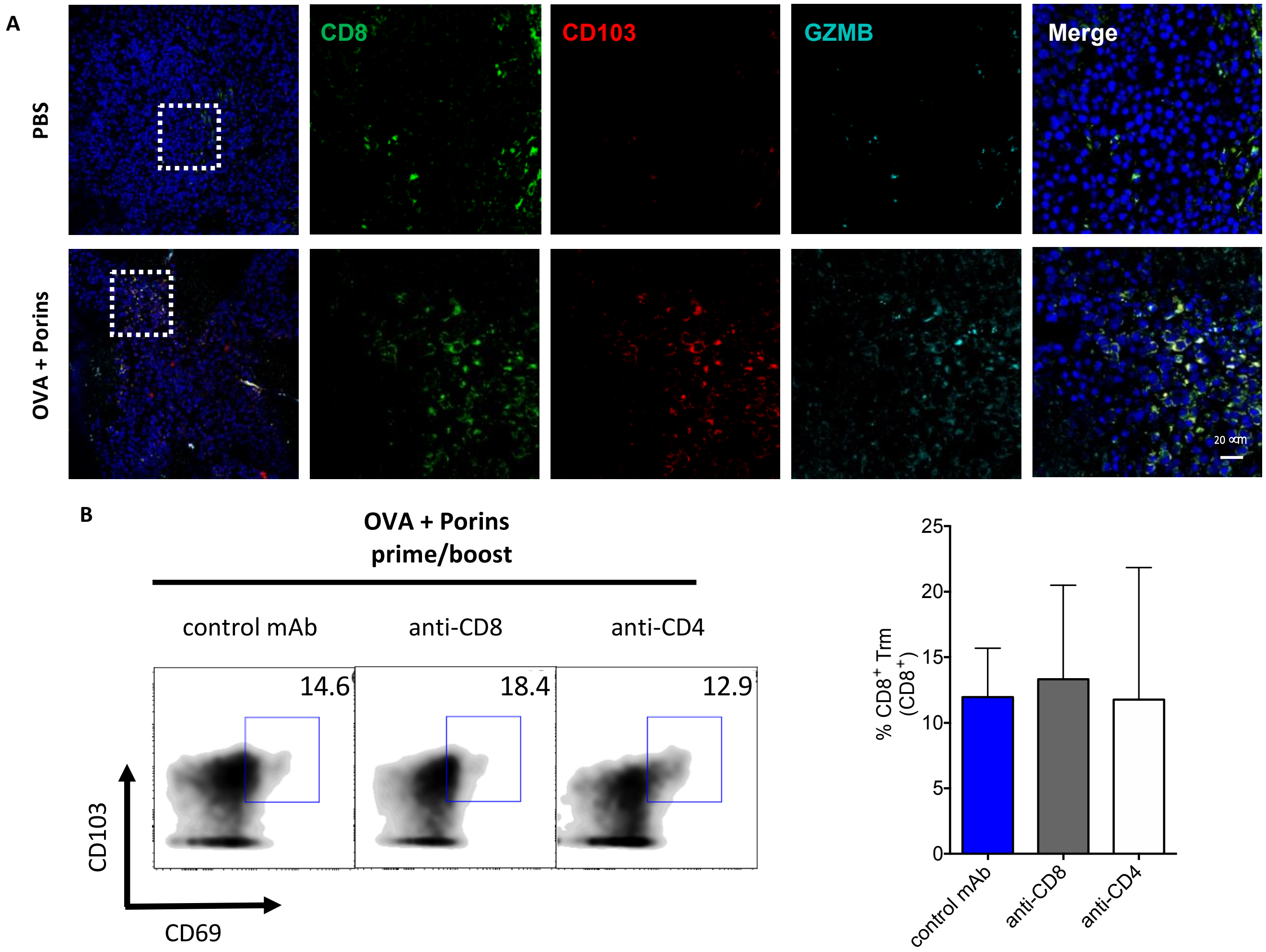

Supplementary Figure 3. Porins-induced functional CD8$^+$ Trm T cells against melanoma. Mice were immunized subcutaneously with OVA + Porins, at days -35 and -28 before MO4 challenge. **(A)** CD103$^+$ (red) CD8$^+$ (green) T cells expressing GZMB (cyan) from tumor of the prime/boost immunization scheme (representative immunofluorescence micrographs from a zoomed area) (Scale bar = 20μm). **(B)** Frequencies of CD8$^+$ Trm TILs of the prime/boost immunization scheme with circulating elimination. Statistical data were pooled from two independent experiments with 2-3 mice per experimental condition.

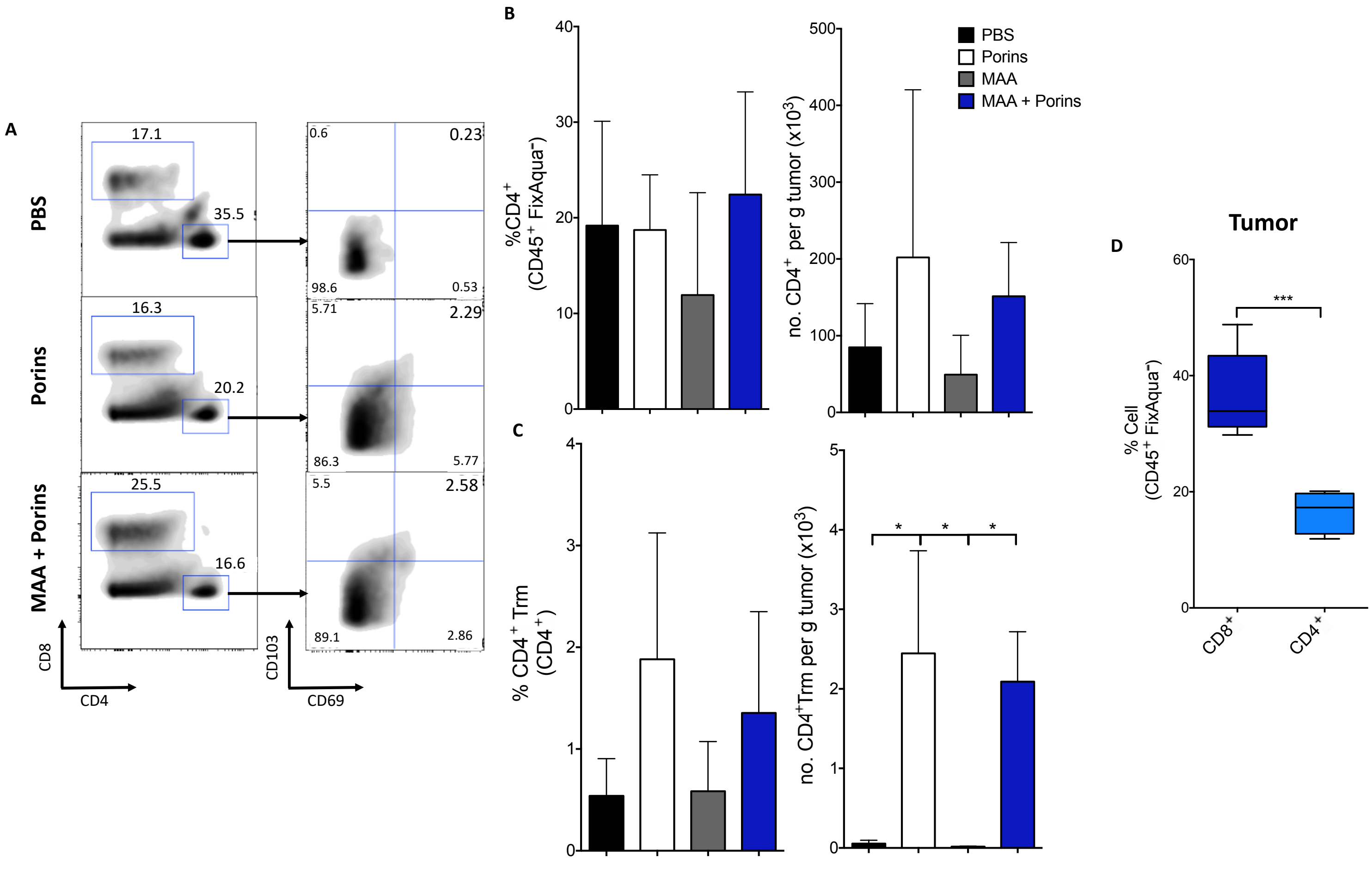

Supplementary Figure 4. Porins-induced CD4+ Trm T cells against native melanoma antigens. Mice were immunized subcutaneously with Porins and TRP-2 and gp100, two melanoma-associated antigens (MAA), at days -10 and -3 before B16-F10 challenge. **(A)** Trm phenotype from CD4+ TILs by flow cytometry. Frequencies and absolute numbers per tumor mass are given of CD4+ T cells **(B)** and CD4+ Trm T cells **(C)**. **(D)** The percentage of CD8+ CD4+ T cells in the tumor. Statistical data were pooled from two independent experiments with 2-3 mice per experimental condition. * $p < 0.05$, *** $p < 0.001$.

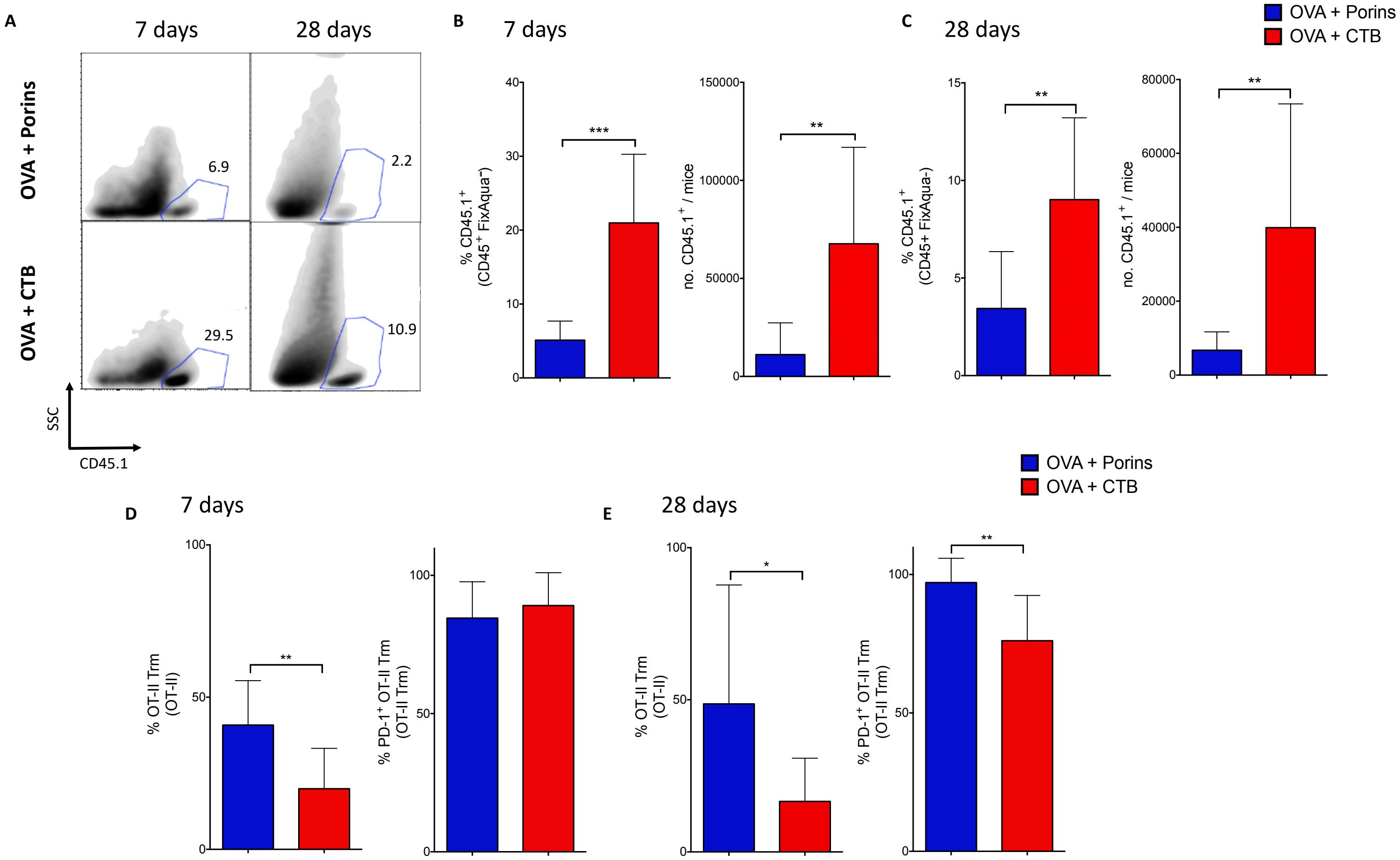

Supplementary Figure 5. Expansion and skin-infiltrating OT-II Trm T cells PD-1^{+} lymphocytes induced by immunizations with Porins or CTB. Mice were adoptively transferred and immunized as in **Figure 2**, comparing the OVA + Porins against OVA + CTB immunization. **(A)** CD45.1^{+} T cell expansion in the skin at 7 and 28 days by flow cytometry. Percentage and absolute number of CD45.1^{+} T cells at day 7 **(B)** and 28 days **(C)** after immunization. Percentage of OT-II Trm T and OT-II Trm PD-1^{+} T cells at day 7 **(D)** and 28 days **(E)** after immunization. Statistical data were pooled from three independent experiments with 3 mice per experimental condition. * $p < 0.05$, ** $p < 0.01$, *** $p < 0.001$.

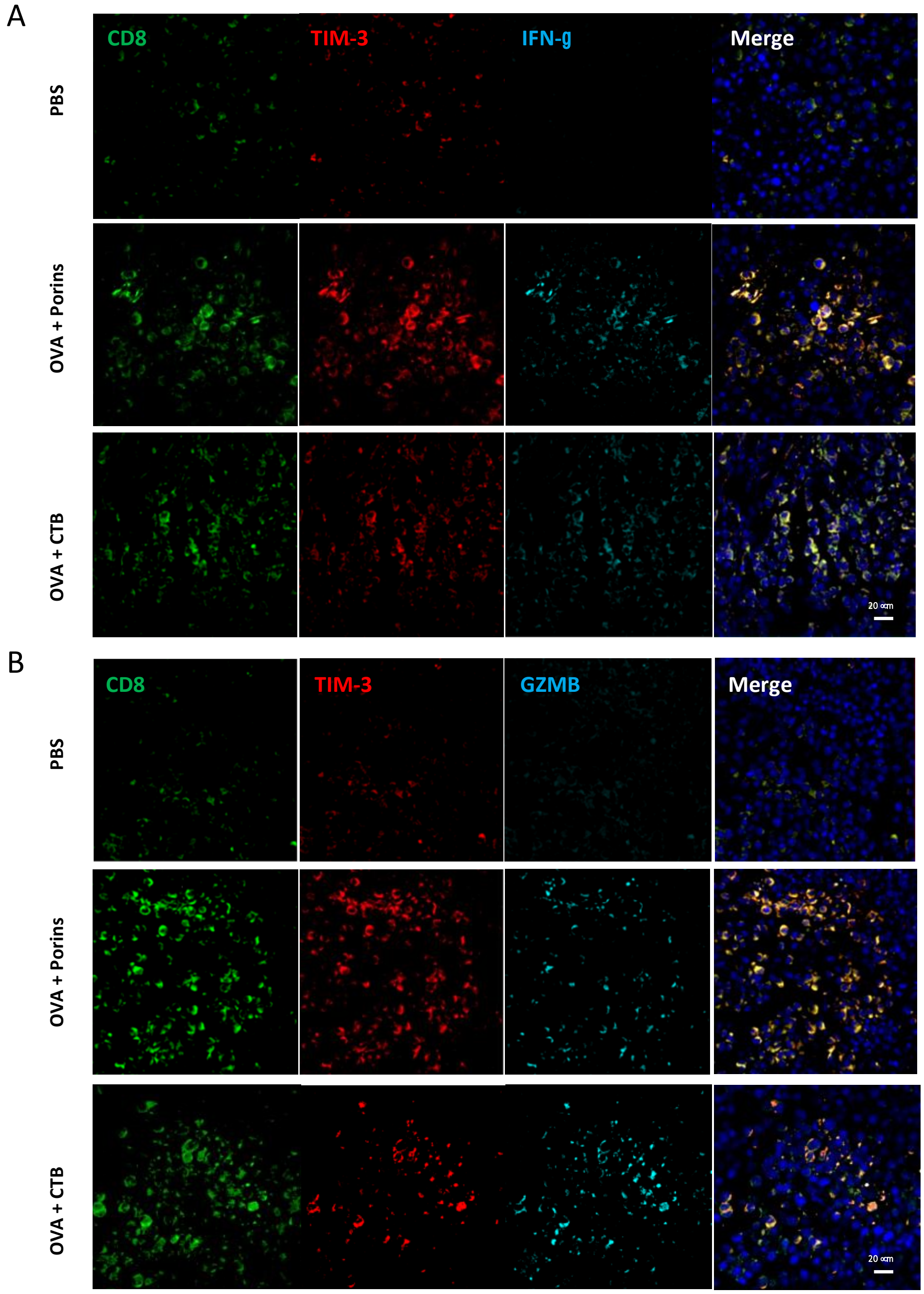

Supplementary Figure 6. Tumor-infiltrating TIM-3^{+} PD-1^{+} CD8^{+} T cells induced by Porins and CTB express IFN-γ and GZMB. Mice were inoculated with MO4 cells and immunized subcutaneously with OVA + Porins, OVA + CTB or PBS control and followed up for 21 days. TIM-3^{+} (red) CD8^{+} (green) TILs expressing IFN-γ (cyan) **(A)** and GZMB (cyan) **(B)** (representative immunofluorescence micrographs from a zoomed area) (Scale bar = 20μm).

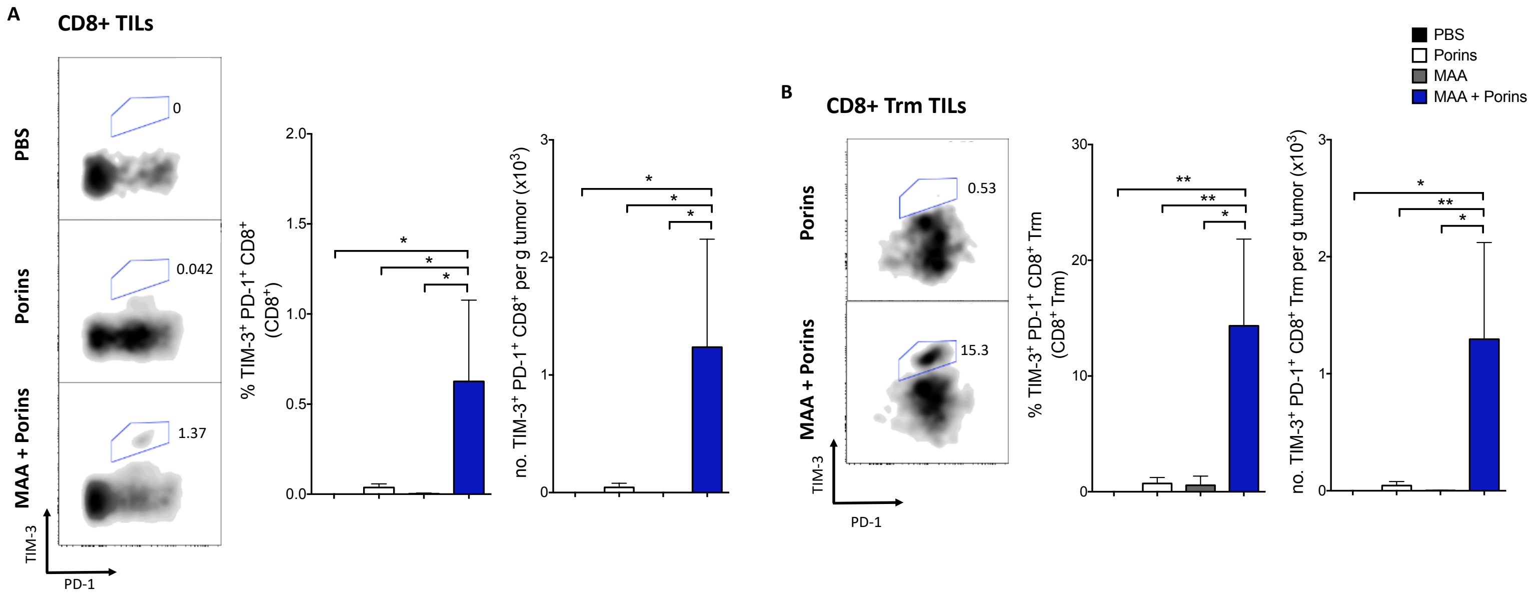

Supplementary Figure 7. MAA + Porins induce formation of TIM-3$^+$ PD-1$^+$ CD8$^+$ and TIM-3$^+$ PD-1$^+$ CD8$^+$ Trm TILs. Mice were immunized subcutaneously with Porins and TRP-2 and gp100, two melanoma-associated antigens (MAA), at days -10 and -3 before B16-F10 challenge. PD-1$^+$ TIM-3$^+$ exhausted phenotypes from CD8$^+$ T cells **(A)** and CD8$^+$ Trm T cells **(B)** by flow cytometry. Frequencies and absolute numbers per tumor mass are shown. Statistical data were pooled from two independent experiments with 2-3 mice per experimental condition. * $p < 0.05$, ** $p < 0.01$.

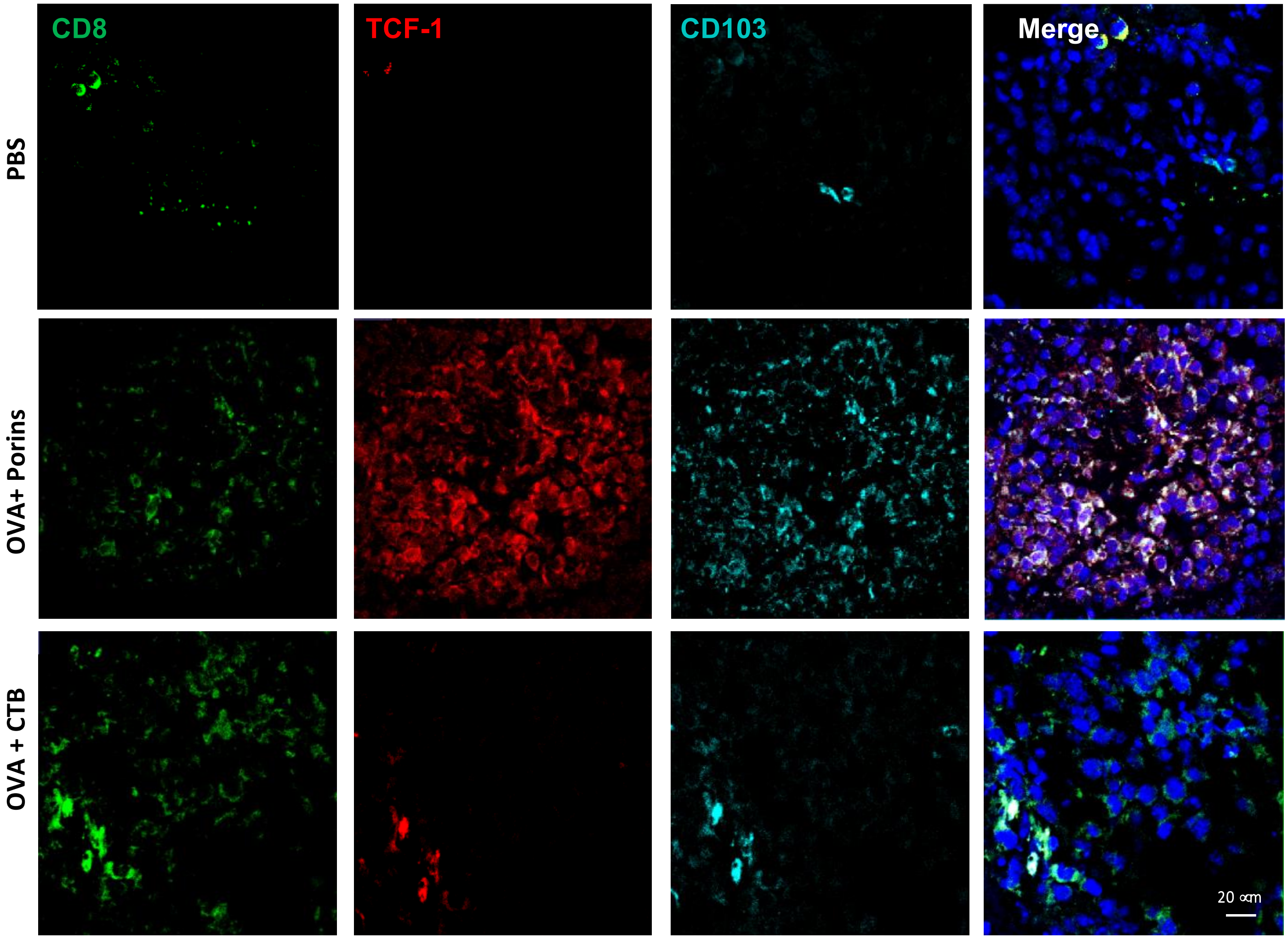

Supplementary Figure 8. Porins induce progenitor exhausted Trm $CD8^+$ TILs in a therapeutic scheme. Mice were inoculated with MO4 cells and immunized subcutaneously with OVA + Porins or OVA+ CTB and followed up for 21 days. TCF-1^+ (red) $CD103^+$ (cyan) $CD8^+$ (green) TILs (representative immunofluorescence micrographs from a zoomed area) (Scale bar = 20μm).

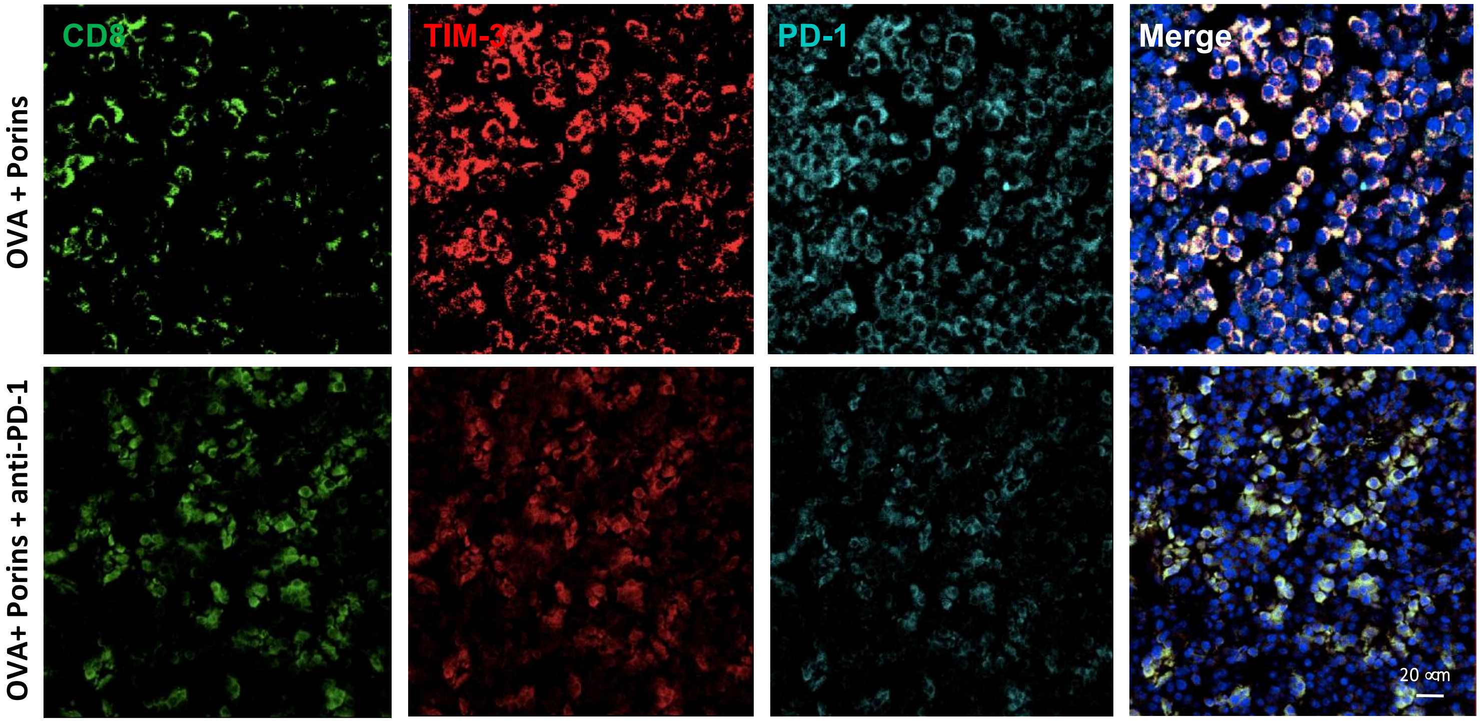

Supplementary Figure 9. TIM-3⁺ PD-1⁺ CD8⁺ TILs induced by Porins combined with PD-1 blockade. Mice were inoculated with MO4 cells and immunized subcutaneously with OVA + Porins, with or without anti-PD-1 mAb, and followed up for 28 days. TIM-3⁺ (red) PD-1⁺ (cyan) CD8⁺ (green) TILs (representative immunofluorescence micrographs from a zoomed area) (Scale bar = 20µm).

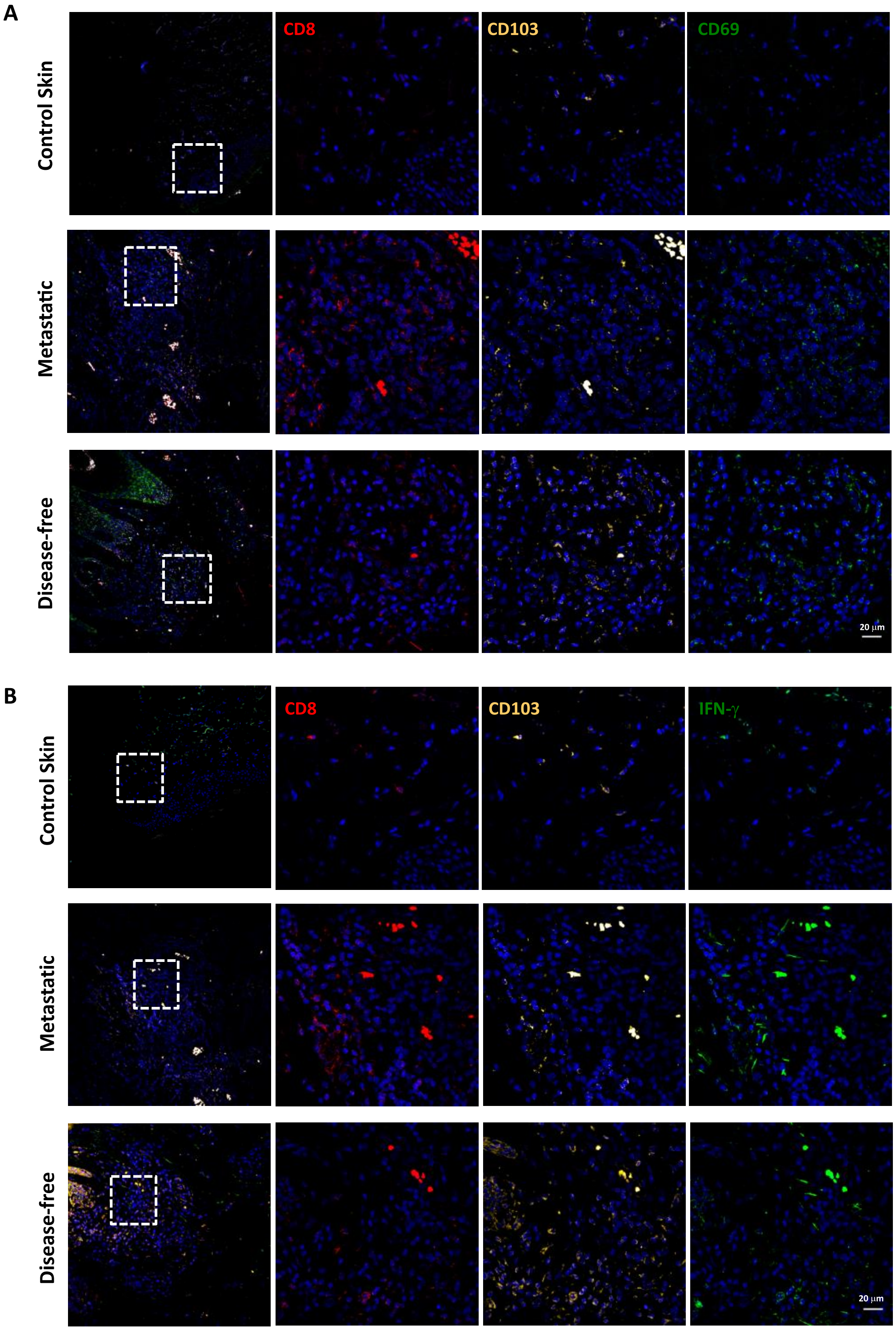

Supplementary Figure 10. IFN-γ^{+} CD8^{+} Trm T cells in the stroma of human melanoma. (A) CD103^{+} (yellow) CD69^{+} (green) CD8^{+} (red) and **(B)** CD103^{+} (yellow) IFN-γ^{+} (green) CD8^{+} (red) T cells from control skin or tumor from metastatic or disease-free melanoma patients (representative immunofluorescence from 20x micrographs [left panels] and 3.4x zoomed images) (Scale bar = 20μm).

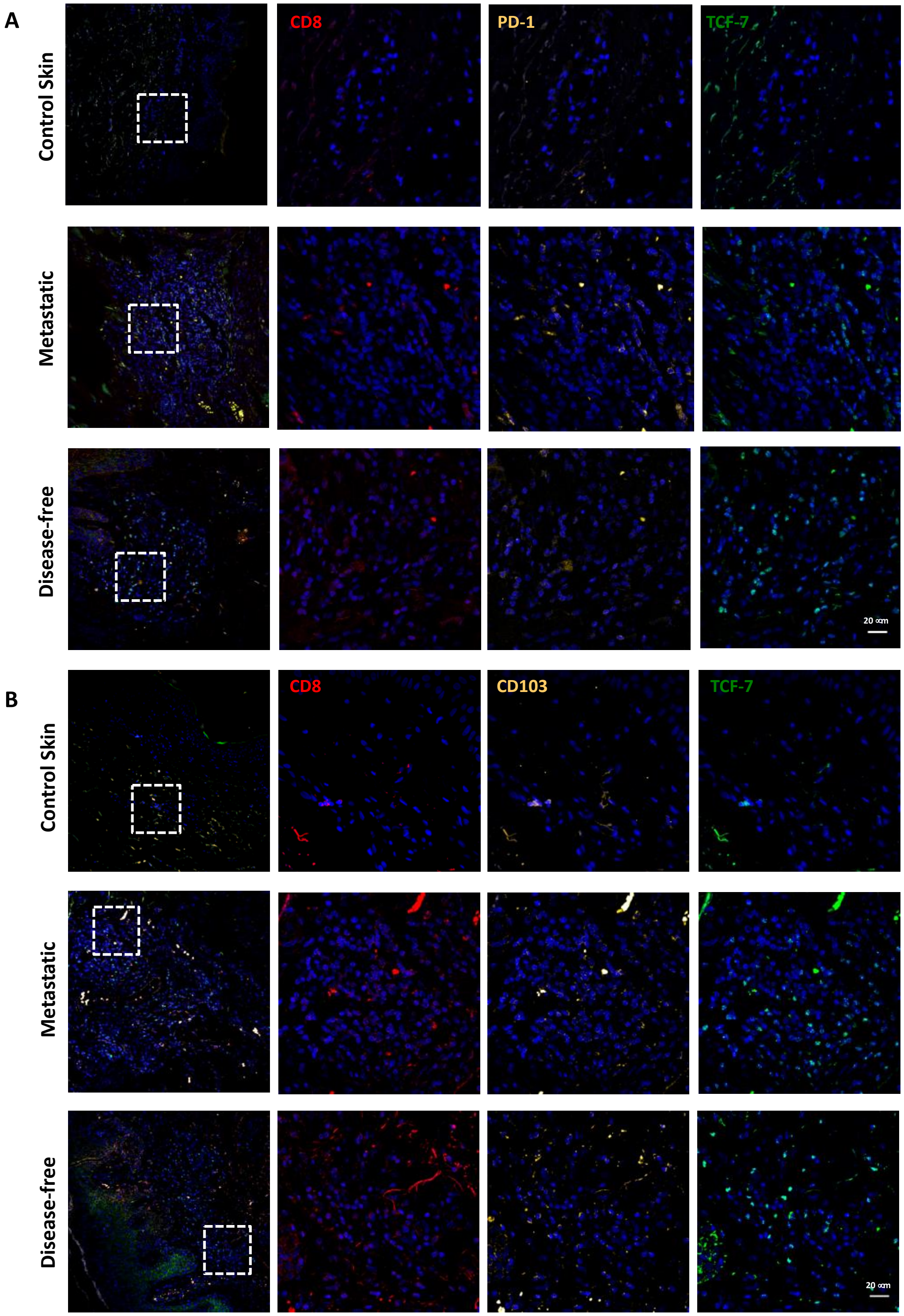

Supplementary Figure 11. Progenitor exhausted CD8$^+$ Trm T cells in the stroma of human melanoma. (A) TCF-7$^+$ (green) PD-1$^+$ (yellow) CD8$^+$ (red) and **(B)** TCF-7$^+$ (green) CD103$^+$ (yellow) CD8$^+$ (red) T cells from control skin or tumor from metastatic or disease-free melanoma patients (representative immunofluorescence from 20x micrographs [left panels] and 3.4x zoomed images) (Scale bar = 20μm).
